# Pt-induced atomic-level tailoring towards paracrystalline high-entropy alloy

**DOI:** 10.1038/s41467-023-36423-1

**Published:** 2023-02-11

**Authors:** Xingjia He, Yu Zhang, Xinlei Gu, Jiangwei Wang, Jinlei Qi, Jun Hao, Longpeng Wang, Hao Huang, Mao Wen, Kan Zhang, Weitao Zheng

**Affiliations:** 1 State Key Laboratory of Superhard Materials, School of Materials Science and Engineering and Key Laboratory of Automobile Materials, MOE, Jilin University, 130012 Changchun, People’s Republic of China; 2 Center of Electron Microscopy and State Key Laboratory of Silicon Materials, School of Materials Science and Engineering, Zhejiang University, 310027 Hangzhou, People’s Republic of China; 3 AECC Beijing Institute of Aeronautical Materials, 81-15 110095 Beijing, China

**Keywords:** Metals and alloys, Glasses, Mechanical properties

## Abstract

Paracrystalline state achieved in the diamond system guides a direction to explore the missing link between amorphous and crystalline states. However, such a state is still challenging to reach in alloy systems in a controlled manner. Here, based on the vast composition space and the complex atomic interactions in the high-entropy alloys (HEAs), we present an “atomic-level tailoring” strategy to create the paracrystalline HEA. The addition of atomic-level Pt with the large and negative mixing enthalpy induces the local atomic reshuffling around Pt atoms for the well-targeted local amorphization, which separates severe-distorted crystalline Zr-Nb-Hf-Ta-Mo HEA into the high-density crystalline MRO motifs on atomic-level. The paracrystalline HEA exhibits high hardness (16.6 GPa) and high yield strength (8.37 GPa) and deforms by nanoscale shear-banding and nanocrystallization modes. Such an enthalpy-guided strategy in HEAs can provide the atomic-level tailoring ability to purposefully regulate structural characteristics and desirable properties.

## Abstract (web summary)

Paracrystalline state is still challenging to reach in alloy systems in a controlled manner. Here, the authors present an atomic-level tailoring route to create paracrystalline Zr-Nb-Hf-Ta-Mo high-entropy alloy through local amorphization induced by atomic-level Pt with negative mixing enthalpy.

## Introduction

Unlike traditional alloys based on one or two principal elements, high-entropy alloys (HEAs) employ five or more elements in relatively high concentrations (5-35 at.%), with an original picture that the maximum configuration entropy engenders the formation of single-phase solid solution^1,2^. Since its inception, this state-of-the-art class of alloys has become the “fertile playground” to explore superior mechanical properties benefits^3,4^, such as high strength, excellent resistance to fatigue and fracture^5,6^, and strong wear resistance^7^, because of their untraditional compositions and chemical structures^8^. In particular, the intrinsic complex interactions among multi-principal components^9^, mainly tied to mixing entropy Δ*S*_*mix*_^10^, mixing enthalpy Δ*H*_mix_^11^, atomic size difference *δ*^12^, electronegativity difference *χ*^13^, and valence electron concentration (VEC)^14^, render the compositional space of high-entropy family vast and uncharted^4,15^; it provides enough opportunities for diverse structural heterogeneity topologically and/or chemically through contemplating elements selection with appropriate numbers, species, and concentrations^16,17^. Generally, enthalpic interactions among principal elements are inevitable to compete with the mixing entropy at relatively low temperatures^9,15^; and then, the high mixing-enthalpy-guided strategy has been come up with for the nano- and/or micro-scale structural heterogeneity in crystalline high-entropy systems, e.g., local chemical order (LCO) in single-phase random solid solution^18^, the solid solution phase embedded by intermetallic precipitates^17,19^, and multiple solid solution phases differing in composition, crystal structure, and lattice constant^20,21^, thereby contributing to boost strength-ductility synergy noticeably. As the regulations above actually depend on the local composition rearrangement via preferentially attracting or repulsing certain atoms due to enthalpic effect^11,22^, introducing atomically dispersed high-enthalpy element may provide a route on the targeted capacity of atomic-level tailoring in the compositional space of the high-entropy system.

Along with the exploration of HEAs, the high negative Δ*H*_mix_ coupled with large *δ* is proposed as the principle for forming high-entropy metallic glasses (HE-MGs)^23^, served as an alternative approach for superior mechanical properties^9,24^. However, HE-MGs, as same as traditional MGs, always suffer from catastrophic failure because the plastic deformation highly concentrates on the single shear band (SB)^25,26^. As a promising strategy, introducing the crystalline medium-range order (MRO) into MGs plays a significant role in the homogeneous plastic flow, instead of the undesirable failure in the form of a dominate SB, as the length scale of MRO is comparable with the shear transformation zones (STZs) carrying plastic flows in MGs^27,28^. In the case of water-cooled and fluxed copper mold casting of the Fe_50_Ni_30_P_13_C_7_ bulk MG^29^, the presence of homogeneously dispersed crystalline MRO nanoclusters has promoted the deformation in multiple SBs rather than a dominate SB, improving the plasticity through the impediment of STZs. In addition, the crystalline MRO regions at the length scale of about 1 nm can be looked upon as precipitates inducing the “precipitation-hardening” effect^30^. Interestingly, a state neither crystalline nor amorphous states, called paracrystalline state^31,32^, which is fully stacked with crystalline MRO mingled with a little disordered matrix, has been recently synthesized in the diamond system^33^. Such a paracrystalline state fills up the gap between amorphous and crystalline states, enduing the paracrystalline diamond with unprecedented combinations of mechanical properties and oxidation resistance. Inspired by such a paracrystalline state in the covalent material system, it is promising to explore the paracrystalline state in metallic materials which may make full use of the aforementioned merits of crystalline MRO in MGs. However, fabricating paracrystalline metallic materials is still facing huge challenges.

Notably, crystallite-containing MGs have been widely produced by inducing the precipitation of nanocrystals and/or MRO during either quenching or secondary treatment^34^. One strategy is simultaneously manipulating the alloy component and cooling rate to trigger partial crystallization during the fabrication process of MGs^29^; the other is the secondary treatments of MGs containing thermal-/deformation-induced crystallization, such as annealing, plastic deformation, and high-pressure torsion^35,36^. Of these, the regulation of thermodynamic parameters is crucial to achieving crystallite-containing MGs. Yet, under the thermodynamic control, it is difficult to achieve the uniform and size-controllable precipitation of crystalline phase in MGs because of the complexity of crystallization processes^37^, particularly for a paracrystalline state that is closely associated with the accurately uniform precipitation of the crystalline MRO but devoid of long-range order (LRO). Subsequently, the quest for an effective approach to synthesizing paracrystalline alloy materials is much-needed. If local disordered clusters are uniformly introduced into relatively large-sized grains, the long-range topologically ordered state of the original grains can be broken and separated into the desired crystalline MRO patches as paracrystallites. In recent research, Wu et al.^38^ have demonstrated that in the Cr-Fe-Co-Ni HEA the uniform introduction of the glass-forming elements B and Si, tending to diffuse and gather at grain boundaries for about 1nm-thickness amorphous tissue, has yielded a nanocrystal-glass dual-phase high-entropy composite during the co-sputtering process. In addition, in certain alloy systems (i.e. Mg_49_Cu_42_Y_9_^39^, Al_92_Ni_2_Y_6_^40^), element separation can be directly activated during the sputtering process to form nanocrystal-glass dual-phase nanocomposite metals. Based on Δ*H*_mix_ and atomic radius mismatch, materials selection rules have been proposed by J.D. Schuler and T.J. Rupert^41^ to introduce amorphous complexion into grain boundaries in binary metallic alloys (Cu-Hf, Cu-Zr systems) during co-sputtering. It has been demonstrated in the above researches that incorporating foreign glass-forming elements into certain alloy systems provides a possibility for nanostructure manipulation by introducing grain-boundary amorphous tissue. If in a special alloy system, the added certain foreign atoms have a strong ability to induce the conversion of the surrounding ordered lattice into topologically disordered groups, the amorphization-targeted foreign atoms would trigger the formation of local disordered groups which homogeneously separate crystals into tiny paracrystallites.

**Fig. 1:**
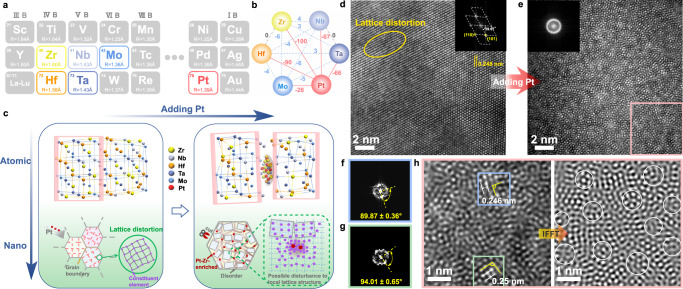
Illustration of design strategy and experimental characterization of the structural evolution from severe-distorted HEA to paracrystalline HEA. **a**, **b** Atomic size and mixing enthalpy of the selected elements for designing the desirable paracrystalline structure in HEA. The gray and blue dotted lines in (**b**) represent the zero and near-zero mixing enthalpy value between constituent elements, respectively. The red lines in (**b**) represent the relatively much larger and more negative mixing enthalpy between Pt and other elements. **c** Schematic diagram of the “atomic-level tailoring” process that the induced disordered groups, driven by foreign Pt atoms, separate the severe-distorted crystalline structure into the paracrystalline structure dominated by crystalline medium-range order (MRO) (marked as pink boxes). Atomic bonding in disordered groups not shown in the diagram. **d**, **e** HRTEM images for the severe-distorted Zr_16_Nb_14_Hf_22_Ta_23_Mo_25_ HEA and the paracrystalline Zr_15_Nb_14_Hf_22_Ta_22_Mo_24_Pt_3_ HEA, respectively. Lattice distortion in Zr_16_Nb_14_Hf_22_Ta_23_Mo_25_ HEA is marked as yellow circle. Insets in (**d**) and (**e**) are the corresponding FFT patterns. **f, g** FFT patterns corresponding to the blue and green squares (1.452 × 1.452 nm^2^) in (**h**), the well-defined bright spots with inclusive angles of around 90°, deriving from the corresponding lattice fringes, suggest the existence of crystalline MRO motifs. **h** Enlarged HRTEM in (**e**) (marked as the pink square) and the corresponding inverse FFT image suggest that the ubiquitous and discernible crystalline MRO motifs are separated by disordered groups (marked as white circles).

In this work, the severe-lattice-distorted crystalline Zr–Nb–Hf–Ta–Mo high-entropy alloy with the atomic radius between 160.25 pm and 136.26 pm (Fig. 1a and Supplementary Table 1) is chosen as the base system; subsequently, utilizing the growth characteristics of co-sputtering, a typical “layer-by-layer” model based on “down-up” strategy, the high-negative-enthalpy foreign Pt atoms are continuously and uniformly introduced into growth front of Zr–Nb–Hf–Ta–Mo HEA, ensuring these Pt atoms atomically dispersed in the HEA lattice sites. The atomic-level intracrystalline Pt atoms may disturb the coordination of surrounding atoms and form favorable Pt–Zr pair due to its Δ*H*_mix_ up to −100 kJ/mol, further triggering local disorder surrounding Pt atoms when the highly negative enthalpy encounters with inherent severe lattice distortion. As a result, based on enthalpy-guided strategy, Pt–Zr-enriched amorphous groups surrounding foreign Pt atoms successfully separate severe-lattice-distorted Zr–Nb–Hf–Ta–Mo grains into crystalline MRO, forming the paracrystalline HEA. It provides a fruitful “menu option” for tailoring atomic-level complexity, controlling MRO in high-entropy families, and opening more accesses for the optimization of mechanical properties.

## Results

### Paracrystalline HEA

In this work, the fragmentizing-grain strategy is proposed to implement paracrystalline HEA completely consisting of crystalline MRO motifs, whose success strongly relies on the atomic-level tailoring ability through the introduced local disorder configuration. For the wishful ability to drive local glass transition stimulated by foreign high-enthalpy atoms, the large *δ* is one of the necessary indicators widely testified in HE-MGs^24,42^. Therefore, crystalline HEA with inherent high-lattice distortion may provide a large and ubiquitous atomic size mismatch that is convenient to the order-to-disorder transition.

**Fig. 2:**
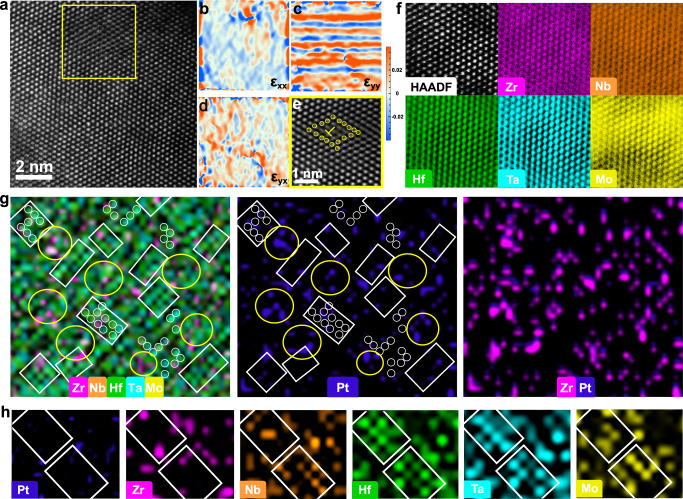
Aberration-corrected TEM imaging and mapping of element distributions in the Zr_16_Nb_14_Hf_22_Ta_23_Mo_25_ and Zr_15_Nb_14_Hf_22_Ta_22_Mo_24_Pt_3_ HEAs, respectively. **a**–**d** Atomic-resolution HAADF image of Zr_16_Nb_14_Hf_22_Ta_23_Mo_25_ HEA and the corresponding atomic strain maps of horizontal normal strain (ε_xx_), vertical normal strain (ε_yy_) and shear strain (ε_yx_), suggesting the severe lattice distortion. **e** The inverse FFT (IFFT) image, corresponding to the area of the yellow square in (**a**) suggests the Burgers circuit encircling the dislocation (marked as “⊥”, the atoms involved are marked with yellow circles). **f** HAADF image of the atomic structure of Zr_16_Nb_14_Hf_22_Ta_23_Mo_25_ HEA, taken with the $[\bar{1}11]$ zone axis, and the EDS maps for individual elements of Zr, Nb, Hf, Ta and Mo, manifesting the relatively homogeneous distribution of each constituent element. **g** For Zr_15_Nb_14_Hf_22_Ta_22_Mo_24_Pt_3_ HEA, elemental EDS map of five constituent elements (Zr, Nb, Hf, Ta, Mo), the individual EDS map of Pt element, and elemental EDS map of Zr and Pt elements in the same location. The ordered atomic arrangement marked by white squares is always located at the Pt-free region while the disordered groups marked by yellow circles are closely linked to the appearance of Pt atoms; the small white circles also reveal the existence of ordered clusters. Pt and Zr atoms tend to appear in the same position, indicating the formation of Pt–Zr-enriched disordered groups. **h** The EDS maps with larger magnification for individual elements of Zr, Nb, Hf, Ta, Mo, and Pt. The white squares represent the Pt–Zr-depleted ordered areas.

Lately, the severe lattice distortion has been introduced into the body-centered cubic (bcc) Nb–Ta–Ti–V HEA by adding a nearly equal amount of element Zr with a larger atomic radius than other constituent elements through the cast and the subsequent long-term treatment at 1200°C^12^, and such distorted lattices verified by Lee et al. from both theory and experiments are uniformly distributed rather than localized in Nb–Ta–Ti–V–Zr HEA. In this work, the high-lattice-distorted bcc Zr_16_Nb_14_Hf_22_Ta_23_Mo_25_ HEA is attained by co-sputtering the large-atomic-radius Zr and Hf targets and relatively small-atomic-radius Nb, Ta, and Mo targets. The representative columnar characteristic widely observed during the sputtering process appears in the Zr_16_Nb_14_Hf_22_Ta_23_Mo_25_ HEA (Supplementary Fig. 1a), in which the columnar grains are well crystallized in the bcc stacking type with the lattice spacing of around 0.250 nm, as ascertained by the high-resolution transition electron micrography (HRTEM) and the corresponding Fast Fourier transform (FFT) patterns (Fig. 1d and Supplementary Fig. 1b), which is consistent with the sharp diffraction peak in XRD pattern (Supplementary Fig. 2). Despite the grains exhibiting the topologically LRO evidenced by the continuous lattice fringes, there exists the severe lattice distortion arising from the large-atomic size mismatch (Supplementary Table 1), in which all atoms involved slightly deviate from their ideal position and form twisty lattice fringes even yield few dislocations or dislocation dipoles, as shown in the atomic-resolution high-angle annular dark-field (HAADF) imaging (Fig. 2a). The atomic-scale element distribution in the Zr_16_Nb_14_Hf_22_Ta_23_Mo_25_ HEA is further investigated in the corresponding energy-dispersive X-ray spectroscopy (EDS) maps (Fig. 2f). The brightness of each colored spot is related to the make-up of the atomic column, which represents the local content of each element^43^. It can be seen that in Zr_16_Nb_14_Hf_22_Ta_23_Mo_25_ HEA, the constituent elements are uniformly distributed which is ensured by the high value of Δ*S*_mix_ (13.18 J/K mol) for the present high-entropy system and the low values of Δ*H*_mix_ among principal elements (Supplementary Table 2, 3). The severe lattice distortion in the highly crystalline Zr_16_Nb_14_Hf_22_Ta_23_Mo_25_ HEA with atoms homogeneous distributed is further demonstrated in the strain maps which are generated by the GPA method with color contours directly illustrating local strains (Fig. 2b–d). Defining the *x*-axis parallel to [101] and the *y*-axis parallel to $[12\bar{1}]$, the calculated strain fields with the red (blue) regions having tensile (compressive) strains are shown in Fig. 2b–d. There exist alternating distributions of large compressive and tensile stress fields along with different directions, with the strain variation ranging from −0.05 % to +0.05 %. Such substantial atomic-level strain fluctuations mean the ubiquitous and severe local strain^8^, which is induced by the atomic-scale lattice distortion that each atom involved experiences different-atomic-radius neighbor atoms^44^ when the fundamental lattice arrangement remains. In addition, the existence of dislocation enlarges the local tensile/compression state in ε_xx_ (Fig. 2b). Much has been said that the severe local strain and the associated large strain energy^45^ caused by large *δ* will promote the lattice instability and be prone to rearrange atomic configuration under external stimuli^46,47^. Furthermore, it has been manifested by the shock compression experiment that the lattice distortion existing in the medium-entropy alloy (MEA) can facilitate the process of amorphization by reducing the energy barrier of amorphization^48^. Taken as a whole, the expectant high-crystalline Zr_16_Nb_14_Hf_22_Ta_23_Mo_25_ HEA coupled with the severe lattice distortion has been obtained, which will be served as the prototype for the following atomic-level tailoring.

Notably, in contrast to the Pt-free HEA with well-defined long-range translationally-ordered atomic stacking, the incorporation of trace amounts of Pt atoms leads to the complete loss of long-range ordered packing configuration in the HRTEM image (Fig. 1e) and the appearance of the diffuse diffraction halo in the FFT pattern associated with the common amorphous feature (the inset of Fig. 1e). Interestingly, the LRO arrangement in Pt-free HEA is replaced by crystalline MRO motifs in the Zr_15_Nb_14_Hf_22_Ta_22_Mo_24_Pt_3_ HEA, agreeing well with the appearance of significantly broadened hump peak in XRD (Supplementary Fig. 2). To carefully observe the general features and more details, the HRTEM image with 1356 × 1356 pixels in Fig. 1e is divided into 9 sub-images, each having 512 × 512 pixels and corresponding to a region with the dimension of 5.808 × 5.808 nm^2^, and then the associated FFT and inverse Fast Fourier transform (IFFT) analyses are employed (Supplementary Fig. 3). Among these, the typical HRTEM and the corresponding IFFT images shown in Fig. 1h suggest that the Zr_15_Nb_14_Hf_22_Ta_22_Mo_24_Pt_3_ HEA mainly consists of crystalline MRO units below a certain scale (~2 nm), which are uniformly divided by the disordered groups on a near sub-nanometer scale. Since all MRO motifs are always highly distorted that atoms often displace from their exact site preservation^31,33^, the typical inclusive angles (89.87 ± 0.36° and 94.01 ± 0.65°) between FFT “diffraction” spots and the around 0.250 nm lattice fringes from crystalline MRO motifs (Fig. 1f, g) both match with the lattice-distorted bcc crystalline arrangement. Therefore, the uniform-distributed disordered groups caused by the addition of Pt can separate the pristine well-crystallized Pt-free Zr–Nb–Hf–Ta–Mo grains into a large number of crystalline MRO motifs. Amazingly, there exist neither nanocrystals more than 2 nm in size nor disordered regions over several nanometer scales. The results may because the disordered groups existing at the end of crystalline MRO motifs (Fig. 1h) have a significant impact on limiting the scale of crystalline MRO^49^, meantime, the scale of disordered groups is suppressed by the nearby crystalline MRO. Wang et al. provided direct experimental evidence that the icosahedra-like atomic clusters, at both ends or inside ordered atomic structures, can suppress the growth of crystalline embryos^49^.

To testify that the structure in Fig. 1e is entirely composed of crystalline MRO motifs, the autocorrelation function (ACF) analysis commonly reflecting the local order information^50,51^ is employed (Supplementary Fig. 4). The HRTEM image with 1356 × 1356 pixels in Fig. 1e is divided into 144 sub-images, each having 128 × 128 pixels and corresponding to a region with the dimension of 1.452 × 1.452 nm^2^. Supplementary Fig. 4 manifests that the obvious and well-defined fringes almost appear in the ACF patterns for all 144 sub-images, suggesting that crystalline MRO structures exist widely and uniformly. The coincident observations also appear in other regions of the sample (Supplementary Fig. 5), manifesting the highly structural homogeneity. In addition, while the selected area electron diffraction (SAED) pattern (the inset of Supplementary Fig. 6a) exhibits the overall amorphous feature, the inspections on the finer scale unveil that only at ~2 nm scale the well-defined sharply bright spots can be identified. In this case, the well-defined bright spots can only appear at the comparable size with crystalline MRO, but these “diffraction” spots deriving from crystalline MRO motifs in the larger selected area will be rotationally averaged and merged into halo rings^29^. It concludes that Zr_15_Nb_14_Hf_22_Ta_22_Mo_24_Pt_3_ HEA can be fully dominated by the crystalline MRO units below 2 nm scale regarded as paracrystallites, revealing that the desirable paracrystalline HEA is acquired. In the paracrystalline HEA, the disordered groups are necessary to separate adjacent crystalline MRO motifs^31^ and simultaneously provide the favorable configuration to connect these crystalline MRO motifs into the paracrystalline network^52^.

Apparently, the transformation from high-crystalline HEA to paracrystalline HEA is closely associated with the introduction of trace foreign negative-enthalpy Pt atoms. It can be noticed that the values of Δ*H*_mix_ among principal elements, approaching 0, render the uniform distribution of all elements in the Pt-free Zr–Nb–Hf–Ta–Mo system (Fig. 2f). Generally, the highly negative Δ*H*_mix_ represents the strong attraction force between atom pairs^11^, and thus the added Pt element with large and negative Δ*H*_mix_ between other constituents will strongly attract these surrounding unlike atoms (Zr, Nb, Hf, Ta, Mo). Moreover, a large difference in Δ*H*_mix_ exists between the foreign Pt atoms and other constituent atoms, which will increase the individual identity of principal elements and promote the local atomic reshuffling corresponding to the chemical inhomogeneity^8,53^. A Similar phenomenon has been demonstrated in the CrMnFeCoNi Cantor alloy in which the Mn element is replaced by the Pd element to induce the different element affinity and tune element distribution for achieving chemical inhomogeneity^8^. Therefore, the atomic-scale element distributions of Zr_15_Nb_14_Hf_22_Ta_22_Mo_24_Pt_3_ HEA are further investigated in the corresponding spherical aberration EDS maps to ascertain the influence of Pt on the atomic level (Fig. 2g, h). As shown in Fig. 2g, many local areas show the ordered arrangement corresponding to the crystalline MRO and others are disordered areas, agreeing with the results in the IFFT image (Fig. 1h). In contrast to the homogeneous distribution of all elements in the Pt-free HEA, the strong chemical heterogeneity presents in either the crystalline MRO motifs or disordered groups after merely incorporating low-content Pt atoms. As shown in Fig. 2h, ordered Hf and Ta atoms with essentially overlapping signals mostly occupy the lattice sites of crystalline MRO motifs in which Nb and Mo atoms are also involved, whereas Zr atoms are depleted in some of these crystalline MRO motifs. Therefore, the crystalline MRO motifs can be regarded as Hf-Ta-rich compounds with a more stable structure through lowering the local strain energy owing to the depletion of the largest-atomic-sized Zr atoms^46,54^. Additionally, it can be noticed that the signal of Pt atoms basically overlaps with the signal of Zr atoms in the merged maps of Pt and Zr elements (Fig. 2g), indicating that foreign Pt atom tend to preferentially form Pt–Zr pair, agree well with the most negative Δ*H*_mix_ of Pt–Zr pair. As further supported by the core-level X-ray photoelectron spectroscopy (XPS) spectra (Supplementary Fig. 7), the addition of low-content Pt in paracrystalline HEA induces the largest shift toward higher binding energy in Zr *3d* core-level spectrum than other elemental spectra. Furthermore, the strongest signal of Pt atoms can be basically assigned in disordered clusters, forming Pt–Zr-rich disordered groups that can be attributed to the large glass-forming ability (GFA) of Pt–Zr clusters with large-atomic size ratio^54,55^. That is, the added Pt atoms uniformly dispersed in the system are similar to the magnets possessing the strongest attractive force with Zr atoms, provided by the most negative Δ*H*_mix_ of the Pt–Zr pair, which preferentially attract Zr atoms around Pt atoms to form local chemical heterogeneity (Pt–Zr-rich groups), creating the local environment for amorphization: large and negative Δ*H*_mix_ and high-lattice distortion. Considering further, because the atomic interaction in the solid solution alloy is limited to the short range within the nearest neighbor of a central atom^56^, the Pt-induced local environment fluctuation can be only confined within the sub-nanometer scale. This is a coincidence with the observation above that the disordered groups localized between crystalline MRO motifs are confined into the near sub-nanometer scale (Fig. 1h). Actually, some researchers have confirmed that doping foreign elements with the high Δ*H*_mix_ into HEAs can create the local environment around the added atoms for chemical heterogeneity from atomic- to nanometer-level^9,16^. Based on the local chemical heterogeneity achieved by doping appropriate foreign metallic elements, the triumph lies mainly in terms of the introduction of LCO and/or crystalline nanoprecipitates into HEAs^11,57^. However, different from the appearance of LCO and/or crystalline nanoprecipitates in HEAs, the local atomic reshuffling around Pt atoms yields the local chemical heterogeneity and subsequent order-to-disorder transition in this case.

Another noteworthy phenomenon is the formation of a periodic compositionally modulated nano-multilayered structure with alternating Pt-rich/-lean nanolayers in Zr_11_Nb_10_Hf_15_Ta_16_Mo_17_Pt_31_ HEA, as shown in Supplementary Fig. 8. A similar compositionally modulated lateral structure along the growth direction has also been reported in Co-Ag and Fe-Ag films, which has been attributed to the spinodal decomposition deriving from the positive-Δ*H*_mix_-driven spontaneous phase separation from supersaturated solid solutions during the film growth^58,59^. It has been demonstrated that the supersaturated clusters tend to initially adsorb at the growth front during sputtering with a rapid quenching rate^60^, subsequently, the competition between phase separation (spinodal decomposition) and strain energy reduction enables the formation of self-assembled nano-multilayered modulated structures^61^. Accordingly, when the Pt content reaches above ~10 at.%, the absorbed Zr–Nb–Hf–Ta–Mo–Pt clusters at the growth front will enter a supersaturated state, and then the substrate temperature of 300 °C can drive phase separation for forming alternating Pt-rich and Pt-lean nanolayers due to negative Δ*H*_mix_, via continuously “layer-by-layer” growth. In addition, the characteristic of FFT patterns remain invariable when the analyzed area is declined down to the 2 nm scale (Supplementary Fig. 8b), and the disappearance of bright spots corresponding to the crystalline MRO means that the paracrystalline structure in Zr_15_Nb_14_Hf_22_Ta_22_Mo_24_Pt_3_ HEA is broken by the high concentration of added Pt atoms. It seems that the Pt-induced local composition variation dominates the local amorphization, and thus the targeted introduction of local amorphization achieved by the precise control of Pt atoms can accurately and uniformly separate the high-crystalline HEA into paracrystalline HEA.

### Formation mechanism of paracrystalline HEA

Regarding the MGs with the large GFA, Inoue^62^ proposed three empirical formulae: (1) at least three elements; (2) the atomic size ratios above 12%; (3) the large and negative mixing enthalpy. Furthermore, as MG systems are extended into HEAs, both ∆*H*_mix_ and ∆*S*_*mix*_ became vital factors that need to be considered^9,23^, based on the Gibbs-Helmholtz equation. Accordingly, in HEAs the parameter *Ω* representing the competition between ∆*H*_mix_ and ∆*S*_mix_ is further proposed as a judgment of GFA^63^, which can be expressed as:

1 $$\Omega=\frac{{T}_{m}\Delta {S}_{{{mix}}}}{|\Delta {H}_{{{mix}}}|}$$

Here, *T*_*m*_ is the weighted average of the melting temperature for each constituent component, estimated from the law of mixtures. In addition, the *δ* has been served as another indicator to judge GFA, as the large size difference usually favors the glass formation against crystallization for high atomic-packing density^2,64,65^. Therefore, the reported empirical conditions combining *Ω* and *δ* have appeared: *Ω* ≥ 1.1 (meaning *T*_*m*_Δ*S*_mix_ predominant the free energy) and *δ* ≤ 6.6 % commonly yield solid solution phase; otherwise, the relatively smaller *Ω* and larger *δ* tend to the topologically disordered amorphous phase^66,67^.

For Zr_16_Nb_14_Hf_22_Ta_23_Mo_25_ HEA, the values of *Ω* and *δ* are calculated to be 9.37 and 6.38 %, respectively; the former is far away from the threshold value of 1.1 and the latter is approaching the value of 6.6%, which still satisfies the empirical rules for forming the stable solid solution^63,67^. Such judgment does take effect on the Zr_16_Nb_14_Hf_22_Ta_23_Mo_25_ HEA existing as the high-crystalline solid solution, suggested by the HAADF image (Fig. 1d). It can be noticed that the incorporation of only 3 at. % Pt can fully break the high-crystalline solid solution structure and form the paracrystalline HEA. In the case of Zr_15_Nb_14_Hf_22_Ta_22_Mo_24_Pt_3_ alloy, compared to Pt-free counterpart, the respective values of *Ω* and *δ* are calculated to be 2.05 and 6.37 %^63^, with basically unchanged *δ* but a precipitous drop of *Ω* (Supplementary Table 3). Besides *δ*, a lattice-distortion factor is better to reflect the degree of lattice distortion in HEAs and has been calculated by first-principles calculations for Zr–Nb–Hf–Ta–Mo and Zr–Nb–Hf–Ta–Mo–Pt HEAs. The results manifest a significant increment in average lattice distortion by adding Pt into Zr–Nb–Hf–Ta–Mo HEA (Supplementary Fig. 9). Although the value of *Ω* experiences a dramatic decrease dominated by the Pt-induced tremendous variation of ∆*H*_mix_ (Supplementary Table 3), it is higher than the empirical value of 1.1 and still meets the solid-solution forming rules^17^. Nevertheless, in the Zr_15_Nb_14_Hf_22_Ta_22_Mo_24_Pt_3_ HEA there exist a great deal of near sub-nanometer-sized disordered groups localized among paracrystallites (crystalline MRO motifs), which can be attributed to the local composition reorganization around Pt atoms to form Pt–Zr-enriched groups (Fig. 2g, h). Such Pt–Zr-enriched groups induced by foreign Pt atoms can lead to a significant increment of |Δ*H*_mix_| and lattice distortion in these local zones, much larger than the average values calculated from the overall system, due to the preferential formation of Pt–Zr pairs with most negative ∆*H*_mix_ of −100 kJ/mol and large *δ*. Namely, the sharp increment of the calculated average value of |∆*H*_mix_| from 3.86 kJ/mol in the Zr_16_Nb_14_Hf_22_Ta_23_Mo_25_ system to 18.43 kJ/mol in the Zr_15_Nb_14_Hf_22_Ta_22_Mo_24_Pt_3_ system should be mainly contributed by the obvious increment of |∆*H*_mix_| in the local Pt–Zr-enriched groups. These Pt–Zr-enriched groups with large and negative ∆*H*_mix_ should be prone to the formation of deep eutectic. On the one hand, such deep eutectic regions can generally create a thermodynamically favorable landscape retarding the crystal nucleation^41,62^. On the other hand, the Pt–Zr-enriched groups significantly limit the kinetics of the crystallization process due to the difficulty of long-range diffusion for different atoms from a kinetic viewpoint based on deep eutectic. In addition, the large *δ* and most negative ∆*H*_mix_ in the local Pt–Zr-enriched groups can also meet Inoue’s empirical rules to achieve local amorphization conditions and trigger the local order-to-disorder transition, which is consistent with the previous reports that Pt–Zr clusters with large-atomic size ratio possess large GFA^54,55^. Accordingly, the Pt-induced local chemical reshuffling can provide Pt–Zr-enriched local regions with enough large *δ* and negative ∆*H*_mix_ for local amorphization, which should be the kernel to introduce the local disordered groups for homogeneously separating the pristine crystals into paracrystallites.

The microstructure evolution further testifies to the tailoring process induced by different concentrations of Pt element (from 0 at.% to 31 at.%), as displayed in Supplementary Fig. 10. Additionally, it seems that the local amorphization ability in HEA should be dominated by the value of Δ*H*_mix_, between added atoms and original constituents, whose diminution will weaken the local amorphization ability so that the foreign element with high concentration is needed. When the foreign Pt element is replaced by the Au element, with the relatively lower values of binary ∆H_mix_ between Au and other constituent elements, a large number of nanocrystals above 2 nm size are still retained even with the higher concentration of Au incorporated in the Zr_15_Nb_14_Hf_19_Ta_20_Mo_24_Au_8_ HEA (Supplementary Fig. 11). Different from the paracrystalline characteristic in Zr_15_Nb_14_Hf_22_Ta_22_Mo_24_Pt_3_ HEA, the common nanocrystal-glass dual-phase nanostructure appears in the Zr_15_Nb_14_Hf_19_Ta_20_Mo_24_Au_8_ HEA due to the weaker local amorphization ability of Au atoms relative to Pt atoms^66^.

### Mechanical properties of HEA and paracrystalline HEA

**Fig. 3:**
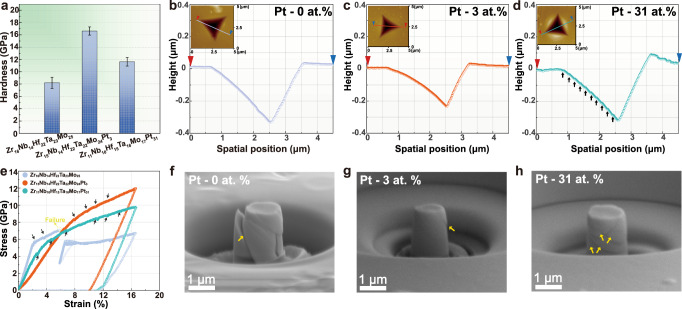
Comparison of mechanical properties of the Zr–Nb–Hf–Ta–Mo HEA with other Pt-bearing HEAs. **a** The hardness results of three HEAs (Pt-free, Pt-3%, Pt-31%). The Pt-3% HEA with the paracrystalline structure exhibits excellent hardness. Error bars represent standard deviation. **b**–**d** Cross-sectional profiles of the indent, along the colored lines of the corresponding inset images (red and blue triangles represent the beginning and end of the profile respectively). A large number of offsets (marked by arrows) corresponding to shear bands (SBs) and the large pileup height around the perimeter of the indent appear in the Pt-31% multilayered amorphous HEA, which are invisible in the Pt-3% paracrystalline HEA. **e** Compression engineering stress-strain curves of three HEAs (Pt-free, Pt-3%, Pt-31%). The pop-in events in the curves are marked by black arrows. **f**–**h** SEM images of the deformed Pt-free, Pt-3%, and Pt-31% micropillars, respectively. The surface offsets on the deformed pillars are marked by yellow arrows.

On the basis of the enthalpy-guided approach, the incorporation of Pt atoms is successful to achieve accurate tailoring of the structure of the high-distorted Zr–Nb–Hf–Ta–Mo system into the paracrystalline feature for Zr_15_Nb_14_Hf_22_Ta_22_Mo_24_Pt_3_ HEA and the multilayered architecture with alternating Pt-lean/rich amorphous nanolayers for Zr_11_Nb_10_Hf_15_Ta_16_Mo_17_Pt_31_ HEA. These nanostructures supplied by Pt addition may activate disparate deformation modes and contribute to the expected excellent mechanical properties, as shown in Fig. 3.

In line with the result that severe lattice distortion in HEA can act as the vital strengthening strategy^12,44^, the lattice-distorted crystalline Zr_16_Nb_14_Hf_22_Ta_23_Mo_25_ HEA has the highest value of hardness (~8.2 GPa) relative to each of the constituent elements (Supplementary Fig. 12). Amazingly, after the formation of paracrystalline structure, the hardness of Zr_15_Nb_14_Hf_22_Ta_22_Mo_24_Pt_3_ HEA has a doubling of increment up to ~16.6 GPa, compared to the Zr_16_Nb_14_Hf_22_Ta_23_Mo_25_ HEA. Furthermore, the hardness in the Zr_11_Nb_10_Hf_15_Ta_16_Mo_17_Pt_31_ HEA decreases slightly when the Pt-lean/rich amorphous nano-multilayers are present, but is still higher than the hardness of Zr_16_Nb_14_Hf_22_Ta_23_Mo_25_ HEA (Fig. 3a). In the further observation on the indentation impressions, as shown in Fig. 3b–d, there are not any cracks existing in the vicinity of the indenter for all three samples. However, in contrast to other HEAs (Pt-free and Pt-3 at.%), several obvious SBs can be identified around the indenter in Zr_11_Nb_10_Hf_15_Ta_16_Mo_17_Pt_31_ HEA owing to its amorphous characteristic. The shear-band-carried deformation mode is further supported by the severe fluctuation on the cross-section profile along with the indenter impression. Such serious local plastic flow carried by SBs yields a large pileup as high as ~90 nm (Fig. 3d). The small outoffsets can be also observed in the Zr_16_Nb_14_Hf_22_Ta_23_Mo_25_ and Zr_15_Nb_14_Hf_22_Ta_22_Mo_24_Pt_3_ HEAs, which should be respectively linked to the local amorphization and the nanometer-sized SBs, exhibiting the more homogeneous plastic flow compared to the stronger localized deformation in Zr_11_Nb_10_Hf_15_Ta_16_Mo_17_Pt_31_ HEA.

Besides the nanoindentation tests, in situ compression tests performed on involved micropillars with a top diameter of ~ 0.9 μm can also show excellent mechanical properties of paracrystalline Zr_15_Nb_14_Hf_22_Ta_22_Mo_24_Pt_3_ HEA, as displayed in the Supplementary Movie 1–3. As shown in the engineering strain-stress curves (Fig. 3e), the lattice-distorted crystalline Zr_16_Nb_14_Hf_22_Ta_23_Mo_25_ HEA has a yield stress of ~5.72 GPa and fails catastrophically at ultimate stress of only ~7.08 GPa (~5.7% strain); it corresponds to the appearance of apparent shearing failure along an angle of ∼ 23 ° to the compressive direction, as displayed in the SEM image of deformed micropillar (Fig. 3f). By contrast, the realization of a paracrystalline structure allows for a significant increase in yield strength up to ~8.37 GPa, with the increasing magnitude about 46% compared to crystalline Zr_16_Nb_14_Hf_22_Ta_23_Mo_25_ HEA. Even if the strain reaches ~16.7%, catastrophic failure can still be avoided in the paracrystalline HEA and the ultimate stress of ~11.98 GPa is reached. Upon further investigation, slight “pop-in” events can be captured on the stress-strain curve of the paracrystalline HEA, which is associated with small shear bands (SBs) on the surface of deformed micropillar^68^ (Fig. 3g). After adding a higher concentration of Pt to obtain a fully amorphous structure in Zr_11_Nb_10_Hf_15_Ta_16_Mo_17_Pt_31_ HEA, its strain-stress curve displays more significant “pop-in” behaviors than paracrystalline HEA, linking to the appearance of severer SBs on the surface of deformed micropillar (Fig. 3h), in agreement with the result of nanoindentation test (Fig. 3d). Besides the paracrystalline HEA, amorphous Zr_11_Nb_10_Hf_15_Ta_16_Mo_17_Pt_31_ HEA also possesses the ability to restrain catastrophic failure; it has a much higher ultimate stress (~9.78 GPa) than crystalline Zr_16_Nb_14_Hf_22_Ta_23_Mo_25_ HEA (~7.08 GPa), but still lower than paracrystalline Zr_15_Nb_14_Hf_22_Ta_22_Mo_24_Pt_3_ HEA (~11.98 GPa). Furthermore, the plasticity of Pt-free/contained HEAs deposited on Ti foils is assessed by the simple bending tests in which all samples are bent to about 45° (Supplementary Fig. 13). Interestingly, the paracrystalline Zr_15_Nb_14_Hf_22_Ta_22_Mo_24_Pt_3_ HEA exhibits high integrity and invisible cracks on the bending surface. By comparison, a large number of long cracks appear along the bending direction and the accompanied partial peeling around the boundary takes place in both Zr_16_Nb_14_Hf_22_Ta_23_Mo_25_ and Zr_11_Nb_10_Hf_15_Ta_16_Mo_17_Pt_31_ HEAs. Above all, the paracrystalline structure (Zr_15_Nb_14_Hf_22_Ta_22_Mo_24_Pt_3_) deforms in the more homogeneous mode and provides integrated merits with higher hardness, higher yield strength and better anti-cracking capacity, compared to either crystalline (Zr_16_Nb_14_Hf_22_Ta_23_Mo_25_) or amorphous (Zr_11_Nb_10_Hf_15_Ta_16_Mo_17_Pt_31_) structure.

### Deformation mechanism of HEA

**Fig. 4:**
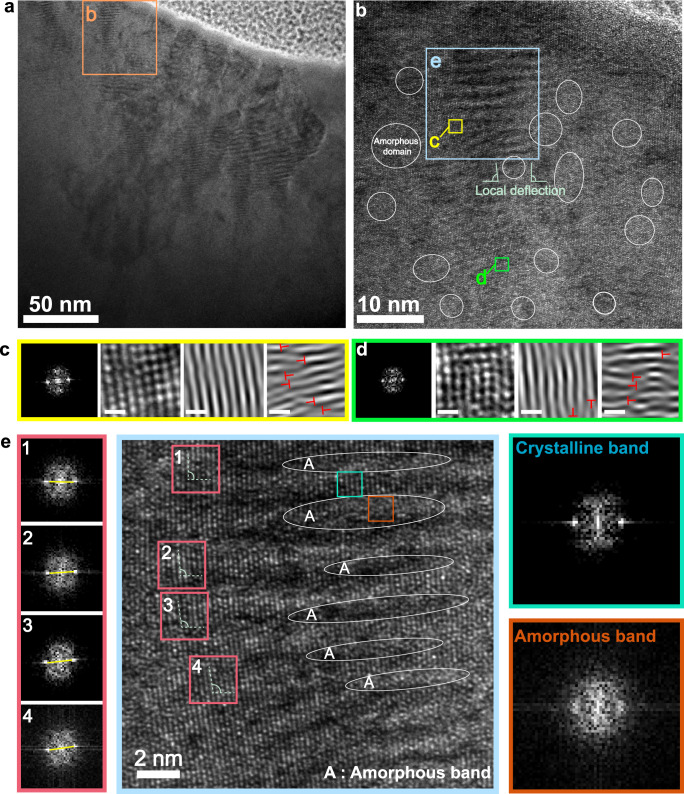
Microstructures of deformation region under indenter in the Zr_16_Nb_14_Hf_22_Ta_23_Mo_25_ HEA. **a** TEM image taken from the cross-sectioned indented region. **b** HRTEM image is taken from the orange square in (**a**) suggesting the existence of lamellar areas and amorphous domains. In addition, local deflections of crystalline lattice are induced by amorphous domains. **c**, **d** FFT pattern and the corresponding two-/one-dimensional inverse FFT images taken from the squared yellow and green area in (**b**), respectively, revealing the existence of numerous dislocations and dislocation dipoles, marked with the symbol ‘⊥’. Scale bar is 0.5 nm. **e** HRTEM image of the lamellar area taken from the blue square in (**b**). The FFT patterns marked by green and reddish-brown squares reveal the crystalline and amorphous features, respectively. The corresponding FFT patterns of pink squares suggest the different degrees of misorientation among these crystalline sub-layers induced by amorphous bands.

To understand the deformation mechanism of lattice-distorted HEA, the microstructure of the deformed regions underneath the indenter is investigated. HRTEM images taken from the cross-sectioned indented region of Supplementary Fig. 14 display a large number of lamellar areas with alternate bands of dark and bright contrast at the tip of the indenter. With a closer inspection of a typical columnar grain subjected to deformation (Fig. 4b, e), a large number of amorphous bands, with 1–4 nm thickness, are introduced along the specific crystallographic direction to periodically partition the original lattice into crystalline/amorphous lamellar areas. The diffuse halo ring of the corresponding FFT pattern (marked by the reddish-brown square in Fig. 4e) further confirms the disordered nature of the amorphous bands as compared to the adjacent crystalline bands. These similar observations of amorphous bands are also induced in the B_4_C materials by either shock-wave loading or nanoindentation^69,70^, in which the crystal collapse along specific orientation for amorphization has been attributed to the violation of Born stability condition caused by anisotropic elastic strains^71,72^. In addition, it can be noticed that the appearance of amorphous bands induces different degrees of misorientation among these crystalline bands, in line with the corresponding FFT patterns (marked by pink squares in Fig. 4e), which should stem from the amorphization-induced pulverization and local deflection of pristine HEA lattice in the process of deformation^73^. Besides the lamellar amorphous bands, the appearance of amorphous domains embedded in the crystalline matrix can also be captured, as shown in Fig. 4b. The identical phenomenon, the appearance of various amorphous configurations above, also happens in other regions below the indentation (Supplementary Fig. 15). The loading-driven amorphization for amorphous bands/domains has been widely observed in various crystalline materials, such as semiconductors (e.g., silicon and germanium), intermetallic (e.g., Ni_3_Al and Ti_3_Al), and certain high-entropy systems (face-centered cubic Cr-Mn-Fe-Co-Ni HEA)^74,75^. The deformation-driven amorphization is usually related to lattice destabilization and derived from the shearing or distortion of crystal lattice which can be strengthened in the present Zr–Nb–Hf–Ta–Mo HEA with severe atomic size mismatch^69,70^.

Generally, deformation-induced amorphization process begins with the formation of crystallographically controlled planar defects^75,76^; especially, the small number of defects in the undeformed crystal (Supplementary Fig. 1c-d) may provide preexisting sites for the nucleation of the amorphous phases in the face of external stimuli^77^. For the deformed region containing amorphous phase, a large number of dislocations and dislocation dipoles, which are regarded as precursors of structural disordering^74,78,79^ and geometrically necessary for accommodating the amorphization process^70^, can be captured in the HRTEM and IFFT images (Fig. 4c, d). The deformation-driven amorphization of Zr–Nb–Hf–Ta–Mo HEA should be mediated by defects (dislocations and dislocation dipoles)^69,73^, which is mainly associated with the shear-induced lattice instability, as proved by previous researches^76,77^. Based on the thermodynamic relationships, when these nonequilibrium defects are incorporated, the internal energy in the crystalline state becomes more than that of the amorphous state and thus the destabilization of crystalline lattice should be considered^75,76^. Under deformation, the defect-containing crystalline phase with higher free energy state would, in principle, transform to a metastable amorphous state to lower its free energy^74,75^, which can be supported by the calculated results (Supplementary Fig. 16). These high-density dislocations and dislocation dipoles in the deformed Zr_16_Nb_14_Hf_22_Ta_23_Mo_25_ HEA should be attributed to the intrinsically high-lattice resistance imposed by the large lattice distortion, providing the obstacles for dislocations travel and significant dislocation-trapping ability^12,74^. In conventional single-phase alloys, the short-range lattice friction is usually exhibited because of the limited number of solute atoms in the matrix. Nevertheless, with different atomic sizes and properties, a plethora of distinct solute atoms in multicomponent HEAs trigger severe lattice distortion accompanied by long-range lattice friction^80,81^. That means the dislocations must travel through the highly distorted solvent lattice in the concentrated multicomponent alloying environment of HEA. Owing to the nearly homogeneous distribution of different solute atoms and the lattice distortion throughout the space, there are no easy ways for dislocation-mobility to bypass the large lattice obstacles, thereby yielding the accumulation of high-density dislocations in the lattice. Additionally, the interlocking of dislocation (dipoles) can also contribute to high pinning resistances, and the back stresses from these trapped dislocations further slowdown the movement of nucleated dislocation (dipoles)^73^. Therefore, in Zr–Nb–Hf–Ta–Mo HEA, these trapped high-density dislocations and dislocation dipoles yield a high free energy state that tends to transform to the amorphous state to lower its free energy.

### Deformation mechanism of paracrystalline HEA

Compared to the severe-distorted crystalline Zr_16_Nb_14_Hf_22_Ta_23_Mo_25_ HEA, the paracrystalline nanostructure mainly consisting of crystalline MRO motifs ensures the double hardness, enhanced yield strength and better anti-cracking capacity. However, these mechanical properties are significantly deteriorated in the full amorphous Zr_11_Nb_10_Hf_15_Ta_16_Mo_17_Pt_31_ HEA with negligible crystalline MRO, which corresponds to the appearance of severe macroscopic SBs on the surfaces of both indenter impression and micropillar. A plethora of crystalline MRO motifs existing in the paracrystalline Zr_15_Nb_14_Hf_22_Ta_22_Mo_24_Pt_3_ HEA should engage and determine the deformation process, subsequently contributing to integrated merits above.

**Fig. 5:**
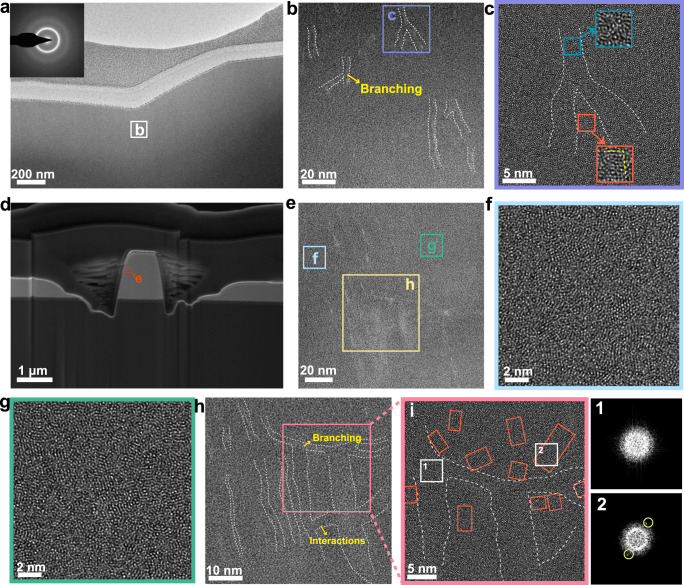
Experimental observations describing deformation behavior in paracrystalline Zr_15_Nb_14_Hf_22_Ta_22_Mo_24_Pt_3_ HEA. **a** TEM image taken from the cross-sectioned indented region shows that there is no macroscopic shear band (SB) can be captured in the deformed paracrystalline HEA. Inset is the corresponding selected area electron diffraction (SAED) image. **b** HRTEM image taken from white square in (**a**), revealing the zigzag feature and branching of nanoscale SBs (marked with white dashed lines). **c** Enlarged HRTEM image in **b** shows the disordered feature inside SB (green square) and the crystalline feature at the bifurcation of SB (orange square). **d** SEM image of the compressed micropillar by focused ion beam (FIB) milling. **e** HRTEM image taken from the orange square in (**d**), suggesting the shear-band-mediated plastic deformation in paracrystalline HEA. **f**, **g** HRTEM images taken from blue and green squares in (**e**), respectively, showing a tendency for crystalline medium-range order (MRO) motifs to grow in size. **h** HRTEM image taken from yellow square in (**e**), manifesting the branching of SBs (marked with white dashed lines) and the interactions between SBs. **i** Enlarged HRTEM image taken from pink square in (**h**). The size of crystalline MRO motifs (marked as orange rectangles) adjacent to nanoscale SBs has clearly increased and some of them have grown into 4–8 nm sized nanocrystals with well-defined lattice fringes, which can be verified by the bright spots in the corresponding FFT pattern (marked as square 2).

To investigate the deformation mechanism of paracrystalline HEA, cross-section TEM specimens are respectively taken from the deformed regions (under nanoindentation, in situ compression and bending tests) by FIB milling. As shown in Fig. 5a, neither obvious SBs nor cracks can be observed beneath the indentation, suggesting a large regime of homogeneous plastic deformation, in line with the results of the AFM image and cross-section profile of indenter impression (Fig. 3c). However, in the Zr_11_Nb_10_Hf_15_Ta_16_Mo_17_Pt_31_ HEA with negligible crystalline MRO, it appears several large SBs corresponding to large strain localization under indentation (Supplementary Fig. 17), manifesting the significance of crystalline MRO responsible for homogeneous deformation. In further exploration of the HRTEM image for paracrystalline HEA, nanoscale SBs with wavy trajectory can be captured in the area beneath the tip of indentation subjected to the largest plastic deformation (marked as the square in Fig. 5a). Similarly, the nanoscale SBs appear in the HRTEM images of bending region (Supplementary Fig. 18). These nanoscale SBs can be easily distinguished from the surrounding matrix as the increased free volume within these small-scaled regions contributes to the relatively lower atomic density for different contrast^82^. Some nanoscale SBs bifurcate into two sub-SBs, probably because the propagation of primary SB is impeded by the local ordering configuration (Fig. 5c)^36,39^. Both the zigzag feature of SBs and the branching of the shearing path contribute to the energy release for homogeneous plastic flow^83,84^.

It can be found that even in the presence of a high density of crystalline MRO motifs, the deformation behavior of paracrystalline HEA is not carried by dislocation motion but by nanoscale SBs. Because the size of crystalline MRO motifs is too small to store dislocation and operate dislocation slip^39,85^. Different from the large-scale SBs in Zr_11_Nb_10_Hf_15_Ta_16_Mo_17_Pt_31_ MG suffering from severe strain localization, the existence of high-population crystalline MRO motifs enables paracrystalline HEA to deform more homogeneously in the form of much-smaller nanoscale SBs. It has been demonstrated by computational analysis that in crystalline-amorphous hybrid alloy systems, the crystalline/amorphous interfaces are prone to act as SB nucleation sites because of structural mismatch^86,87^. For example, the investigation on plastic deformation of an MG embedded with approximately 37% of a bcc phase by molecular dynamics has revealed that the nanocrystalline/amorphous interfaces will serve as SB precursor^87^, which stems from the high potential energy of atoms at the interface and stress concentration induced by the elastic-moduli difference between heterogeneous phases. In addition, finite element calculations on (Cu_60_Zr_30_Ti_10_)_0.95_Ta_5_ crystalline-amorphous alloy system also proved that a number of strain-localized zones prefer to develop at the crystalline/amorphous interfaces to maintain strain compatibility, which subsequently acts as the initiation sites of SBs^85^. As paracrystalline HEA is fully stacked with crystalline MRO separated by disordered groups, the crystalline-MRO-motifs/disordered-groups interfaces are ubiquitous and uniformly distributed. It can be speculated that these high-density interfaces can supply fertile sites throughout the deformed space for the nucleation of nanoscale SBs. A large number of SB nucleation sites in paracrystalline HEA can facilitate the shear delocalization, providing the basis for the development of multiple nanoscale SBs. Therefore, the plastic flow in the paracrystalline HEA distributes globally, compared to the large SBs in Zr_11_Nb_10_Hf_15_Ta_16_Mo_17_Pt_31_ MG.

Additionally, the deformed micropillar of paracrystalline HEA is further investigated by TEM, as shown in Fig. 5d. A large number of nanoscale SBs and sub-SBs can also be found in HRTEM images (Fig. 5e). Besides nanoscale-SB behavior, a tendency for crystalline MRO motifs to grow in size can be observed (Fig. 5f, g, i), which has been also reported in certain SB-mediated deformed systems due to strain effect and/or thermal effect^36,88^. Identifying the SB-affected regions in detail, the size of crystalline MRO motifs adjacent to nanoscale SBs has increased and some of them have grown into ~4–8 nm sized nanocrystals with well-defined lattice fringes (marked as orange squares in Fig. 5i). Compared to the boundary area of nanoscale SBs with partial precipitation and growth of nanocrystals, the central regions of SBs exhibit a more disordered feature, which is verified by the corresponding FFT patterns obtained at the center and edge of SB, respectively (Fig. 5i). This similar phenomenon that nanocrystals preferentially precipitate at the boundary area of SBs has been also observed in Cu-based bulk MGs under uniaxial compression^89^. Simulations have revealed that the highest local temperature increase is located at the shear plane (the central region of SB) which should be the preferred location for heating-induced crystallization to occur^90^. Based on these estimations we can exclude that the more ordered configurations and growth of nanocrystals during deformation stem from the temperature rise. Instead, the nanocrystallization present in this work should be associated with stress-induced atomic displacements^36,90^. Generally, this local rearrangement of small atomic groups as STZ behavior can introduce the free volume and lead to an expansion effect^83^. It has been reported that the dilated state appears inside an SB that is subjected to tensile stress and the regions in the vicinity of SB are relatively subjected to compressive stress^90^. These jammed atoms under the compressive state are prone to redistribution to form an orderly configuration for nanocrystallization, based on potential energy landscape theory^36,90^. Besides the process via diffusive actions of individual atoms, the atomic rearrangement for crystal nucleation and growth under external stimuli can also be explained by the cluster-coupled process that small groups of atoms are stimulated to perform shear-diffusion-transformations^37,91^. With sufficient crystalline MRO motifs accumulated collectively in paracrystalline HEA, the local region involving these crystalline MRO motifs is ready to be pushed to join and expand into nanocrystals under the impact of compressive stress. As present in previous research, the precipitation and growth of nanocrystals are expected to relax stresses in the surrounding matrix and hamper the formation of STZ behavior^92^, namely the process of nanocrystallization competes with the initiation of nanoscale SBs. In addition, the nascent nanoscale SBs generated by the percolation of STZ regions are forced to detour, bifurcate, or terminate owing to the obstacle effect of the adjacent crystalline MRO motifs and nanocrystals. Notably, the deformation in paracrystalline HEA is mainly mediated by the nanoscale SB mode and accompanied by nanocrystallization. The crystalline MRO motifs and growing nanocrystals can serve as obstacles to postpone the formation and propagation of SBs, promote multiple branching of SBs and confine SBs to the nanoscale via shear delocalization; therefore, the macroscopic shear localization, existing in the Zr_11_Nb_10_Hf_15_Ta_16_Mo_17_Pt_31_ HEA, can be almost completely inhibited in the paracrystalline structure filled with crystalline MRO motifs.

To conclude, incorporating a trace amount of Pt element, possessing the large and negative Δ*H*_mix_ between other constituent elements, into the severe-distorted crystalline Zr–Nb–Hf–Ta–Mo HEA can trigger the local composition reconfiguration around Pt atoms to satisfy the local amorphization conditions: the large and negative Δ*H*_mix_ and the high distortion. The disordered groups induced by uniformly distributed Pt atoms can separate the pristine distorted HEA into paracrystallites on atomic-level. Such paracrystalline HEA hampers the shear localization for homogeneous plastic deformation and shows the power on both enhanced hardness and improved plasticity. The proposed “atomic-level tailoring” strategy by introducing disordered groups in a controllable manner can provide enough opportunities to construct the atomic/nano-level structure heterogeneity topologically and/or chemically, accordingly opening up the effective way to elevate the mechanical properties. It can be expected that paracrystalline HEA fabricated by sputtering technology can be used as the protective coating with potential application in the field of machine parts, medical implants, micro-electromechanical systems, etc.

## Methods

### Fabrication of the materials

All high-entropy samples investigated in the present study were synthesized via magnetron co-sputtering ZrNbHfTaMo compound target and Pt or Au target (99.9 at.% purity). The five fan-shaped elemental metallic targets (Zr, Nb, Hf, Ta, Mo) of the same size were spliced together to form the ZrNbHfTaMo compound target with a diameter of 60 mm. The composition of the films was controlled by tuning the sputtering power applied to the Pt target, and a ~150 nm-thickness Ti interlayer was deposited on the Si wafers to improve the adhesion between the alloys and the Si substrates. The target-to-substrate distance was controlled at 70 mm. During the sputtering process, the base pressure was lower than 10^−4^ Pa, and the working pressure was 0.8 Pa through bleeding the Ar gas with a flow of 70 sccm into the chamber when the substrate temperature was maintained at 300 °C. The deposition was carried out for 120 min with an applying current of 450 mA for ZrNbHfTaMo compound target supplied by direct current power and variable sputtering power for the Pt target (0 W, 10 W, 100 W) supplied by radio-frequency power, and the thicknesses for all obtained films are ranging from 2.0 μm to 2.8 μm. In the comparative experiment, the Pt target was replaced with an Au target, and the sputtering power of 20 W was supplied by radio-frequency (RF) power applied to the co-sputter with the ZrNbHfTaMo compound target when other conditions were maintained the same. In addition, to further demonstrate the Pt-induced impact on the structure evolution rapidly and accurately, the compositionally-graded sample containing 8 sub-layers was fabricated on the Al_2_O_3_ wafer. The composition gradient was controlled by tuning the RF sputtering power applied to the Pt target and each HEA layer was sustained at ~250 nm.

### Structural characterization

To ensure the concentration of each element involved, the composition (in at.%) of samples was measured by using Electron Probe Micro Analysis (EPMA, JXA-8230). The cross-sectional TEM samples were prepared with a focused ion beam (FIB) instrument (FEI Strata 400S). Before milling, the contact areas were coated by a protective layer of Au. The final milling voltage/current was sufficiently small to avoid potential crystallization or amorphization. The field emission high-resolution TEM (HRTEM, JEM 2100F) operating at 200 kV was first employed. Then, the JEM-ARM300F double spherical aberration transmission electron microscope was used for further structural characterization at the atomic level. High-resolution STEM imaging and Energy Dispersive X-ray Spectroscopy (EDS) were carried out using a JEM-ARM300F GRAND ARM operated at 300 kV. The phase structures of all samples were determined by X-Ray Diffraction (XRD) using a Bragg-Brentano diffractometer (D8_tools) in ϴ–2ϴ configuration with Cu Kα radiation. Raman spectra were measured on a micro-Raman spectrometer (Renishaw) at the laser power of 0.5 mW, in which the laser with a wavelength of 532 nm was equipped.

### Mechanical tests and characterization

An instrumented nanoindenter (MTS, Nano Indenter XP) equipped with a Berkovich tip was utilized to measure the hardness and modulus of samples, using continuous stiffness mode (CSM) with a maximum displacement of 500 nm and at a strain rate of 0.1 s^−1^ at room temperature. The values of hardness were taken from the indentation depth range of 100–200 nm, less than 10 % of the film thickness, to minimize the contributions of both substrate and surface roughness. Nine points were measured for each sample to get the mean values. Bending plasticity was evaluated by a simple bend test. The morphologies around nanoindentations and on the surface of bend-test samples were examined by scanning electron microscopy (SEM) (JEOL JSM-6700F) and atomic force microscope (AFM, Dimension Icon, Veeco Instruments/Bruker). The cross-sectional samples for indented samples were prepared with a FIB instrument (FEI Strata 400S) and observed via JEM 2100 F HRTEM. In situ compression tests were performed using a Hysitron TI950 nanoindenter with a 5-μm diamond flat punch, under displacement-controlled mode of ~2 nm/s displacement rate. Micropillars for uniaxial micro-compression tests were fabricated using a FIB instrument (FEI Strata 400S). The height of the pillars was ~ 1.8 μm, and the aspect ratios (height/diameter) of the pillars were 2. The cross-sectional TEM sample was prepared by FIB milling.

### First-principles calculations

First-principles calculations were carried out with the Vienna Ab-initio Simulation Package (VASP), using the projector-augmented-wave (PAW) method. The exchange-correlation energy was described with the generalized gradient approximation (GGA) in the Perdew–Becke–Ernzehof (PBE) parameterization. The structural optimizations of Zr–Nb–Hf–Ta–Mo HEA and Zr–Nb–Hf–Ta–Mo–Pt HEA were performed, with a cutoff energy of 520 eV and Monkhorst-Pack k-point grid of 2 × 2 × 2. The chemical disorder of the solid solution was modeled, using the Special Quasi-random Structures (SQS). A cell containing 50 atoms was generated for the Zr–Nb–Hf–Ta–Mo HEA, and Zr–Nb–Hf–Ta–Mo–Pt HEA, respectively. The atomic positions and structural parameters of constructed SQS were optimized with 0.01 Å and an electronic energy convergence criterion of 10^-5 ^eV/atom. The lattice distortion level was evaluated by measuring the bond lengths of the two structures after structural optimization. For calculating the Gibbs free energy, four types of the 80-atom Zr–Nb–Hf–Ta–Mo HEA model with equiatomic ratios are built via SQS. Model I: the crystalline model without defects; Model II: the crystalline model with low-density defects, i.e., one edge dislocation generated by *atomsk* package; Model III: the crystalline model with high-density defects, i.e., two edge dislocations generated by *atomsk* package and several point defects induced by shifting the atom to interstitial site; Model IV: the amorphous model generated by ab initio molecular dynamics after 2500 fs at 3000 K, with cutoff energy of 600 eV. Subsequently, Gibbs free energies of four states are obtained based on 3N phonon vibration frequency calculation.

## Supplementary information

Supplementary Information

Description of Additional Supplementary Files

Supplementary Movie 1

Supplementary Movie 2

Supplementary Movie 3

## Data Availability

The data supporting the key findings of this study are available within the article and its Supplementary Information files. Any further relevant data are available from the corresponding authors on request.
