# Supplementary material for: Pt-induced atomic-level tailoring towards paracrystalline high-entropy alloy
Source: Nat Commun. 2023 Feb 11;14:775. doi: 10.1038/s41467-023-36423-1 (PMC9922268; doi:10.1038/s41467-023-36423-1)
Supplement: Supplementary file 1 — Supplementary Information [file 41467_2023_36423_MOESM1_ESM.pdf]

Supplementary Information for

Pt-induced atomic-level tailoring towards paracrystalline high-entropy alloy

Xingjia He[1], Yu Zhang[1], Xinlei Gu[1], Jiangwei Wang[2], Jinlei Qi[1], Jun Hao[1], Longpeng Wang[1], Hao Huang[3], Mao Wen[1]✉, Kan Zhang[1]✉ and Weitao Zheng[1]✉

[1]State Key Laboratory of Superhard Materials, School of Materials Science and Engineering and Key Laboratory of Automobile Materials, MOE, Jilin University, Changchun 130012, People's Republic of China

[2]Center of Electron Microscopy and State Key Laboratory of Silicon Materials, School of Materials Science and Engineering, Zhejiang University, Hangzhou 310027, People’s Republic of China

[3]AECC Beijing Institute of Aeronautical Materials, Beijing81-15 110095, China.

Table of content

I. Supplementary figures and tables

Supplementary Fig. 1 | Structural characterization of the $Zr_{16}Nb_{14}Hf_{22}Ta_{23}Mo_{25}$ HEA

Supplementary Fig. 2 | XRD patterns of three HEAs ($Zr_{16}Nb_{14}Hf_{22}Ta_{23}Mo_{25}$, $Zr_{15}Nb_{14}Hf_{22}Ta_{22}Mo_{24}Pt_3$ and $Zr_{11}Nb_{10}Hf_{15}Ta_{16}Mo_{17}Pt_{31}$)

Supplementary Fig. 3 | FFT and the corresponding inverse FFT (IFFT) images taken from the divided HRTEM image in Fig. 1e

Supplementary Fig. 4 | Representative autocorrelation function (ACF) statistical analysis of Fig. 1e

Supplementary Fig. 5 | The structural characterization and analysis of paracrystalline $Zr_{15}Nb_{14}Hf_{22}Ta_{22}Mo_{24}Pt_3$ HEA

Supplementary Fig. 6 | The changing FFT characteristics with the zoom-out selected area in the HRTEM image of $Zr_{15}Nb_{14}Hf_{22}Ta_{22}Mo_{24}Pt_3$ sample

Supplementary Fig. 7 | XPS core level spectra of Zr *3d*, Nb *3d*, Hf *4f*, Ta *4f*, Mo *3d*, Pt *4f* performed on three HEAs (Pt-0 at.%, Pt-3 at.%, Pt-31 at.%).

Supplementary Fig. 8 | Structural characterization and illustration of $Zr_{11}Nb_{10}Hf_{15}Ta_{16}Mo_{17}Pt_{31}$ HEA

Supplementary Fig. 9 | Predictions of the interatomic distances for Zr-Nb-Hf-Ta-Mo and Zr-Nb-Hf-Ta-Mo-Pt HEAs via first-principles calculations.

Supplementary Fig. 10 | Microstructure evolution of the compositionally-graded sample

Supplementary Fig. 11 | Structural characterization of $Zr_{15}Nb_{14}Hf_{19}Ta_{20}Mo_{24}Au_8$ HEA

Supplementary Fig. 12 | Hardness of $Zr_{16}Nb_{14}Hf_{22}Ta_{23}Mo_{25}$ HEA and each corresponding constituent sample

Supplementary Fig. 13 | Simple bending tests

Supplementary Fig. 14 | HRTEM images of the deformed region under indenter in

$Zr_{16}Nb_{14}Hf_{22}Ta_{23}Mo_{25}$ HEA

Supplementary Fig. 15 | Microstructures of overall deformation region containing differently oriented grains under indenter in the $Zr_{16}Nb_{14}Hf_{22}Ta_{23}Mo_{25}$ HEA

Supplementary Fig. 16 | A schematic diagram of free energy level for Zr-Nb-Hf-Ta-Mo HEA in different deformation states

Supplementary Fig. 17 | Microstructures of deformation region under indenter in the $Zr_{11}Nb_{10}Hf_{15}Ta_{16}Mo_{17}Pt_{31}$ HEA

Supplementary Fig. 18 | Microstructures of bent region in the paracrystalline $Zr_{15}Nb_{14}Hf_{22}Ta_{22}Mo_{24}Pt_3$ HEA

Supplementary Table 1 | Atomic radius of each element involved in the present HEAs

Supplementary Table 2 | The values of $\Delta H_{\{AB\}}^{mix}$ (kJ/mol) for atomic pairs between the consistent elements involved in the present HEAs

Supplementary Table 3 | Calculated parameters ΔH_{mix}, ΔS_{mix}, δ, and Ω by average method for $Zr_{16}Nb_{14}Hf_{22}Ta_{23}Mo_{25}$, $Zr_{15}Nb_{14}Hf_{22}Ta_{22}Mo_{24}Pt_3$, $Zr_{11}Nb_{10}Hf_{15}Ta_{16}Mo_{17}Pt_{31}$, and $Zr_{15}Nb_{14}Hf_{19}Ta_{20}Mo_{24}Au_8$ HEAs, predicting the Pt/Au-induced structural evolution

II. Supplementary References

I. Supplementary figures and tables

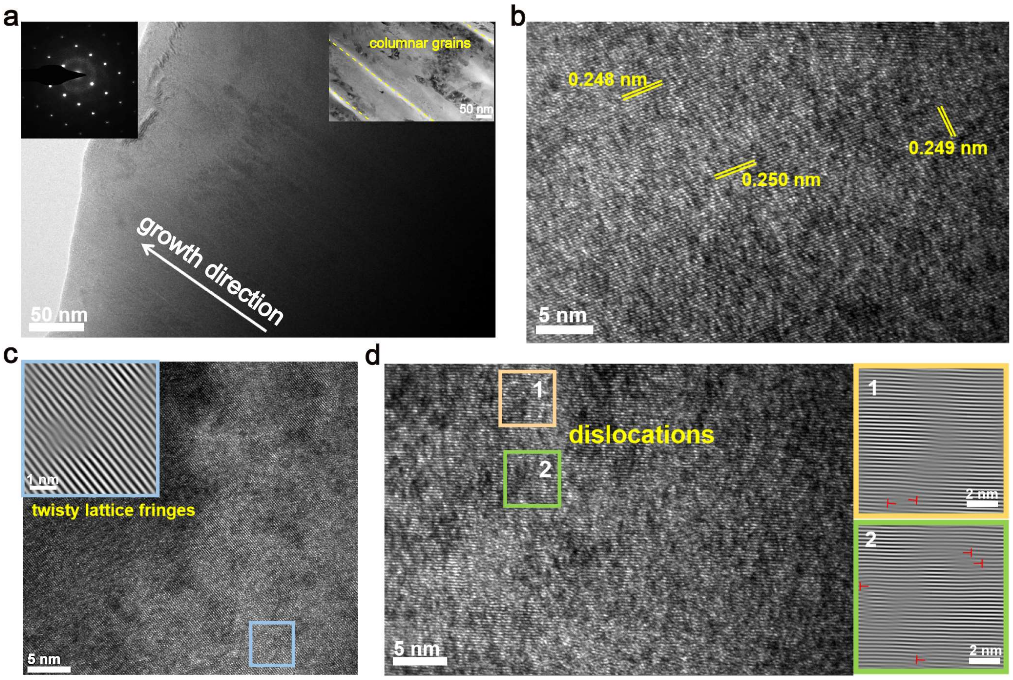

Supplementary Fig. 1 | Structural characterization of the $Zr_{16}Nb_{14}Hf_{22}Ta_{23}Mo_{25}$ HEA. a, TEM image revealing the characteristic of columnar grains with a well crystalline state (shown in the diffraction pattern and atomic-resolution high-angle annular dark-field image in the inset). **b**, The lattice fringes with a lattice spacing of ~0.250 nm in the HRTEM image are assigned to the (110) plane of the body center cubic (bcc) phase. **c**, **d**, HRTEM images and the corresponding inverse Fast Fourier transform (IFFT) images taken from the squared area, suggesting the existence of twisty lattice fringes, dislocations and dislocation dipoles. Dislocations are marked with the symbol "⊥".

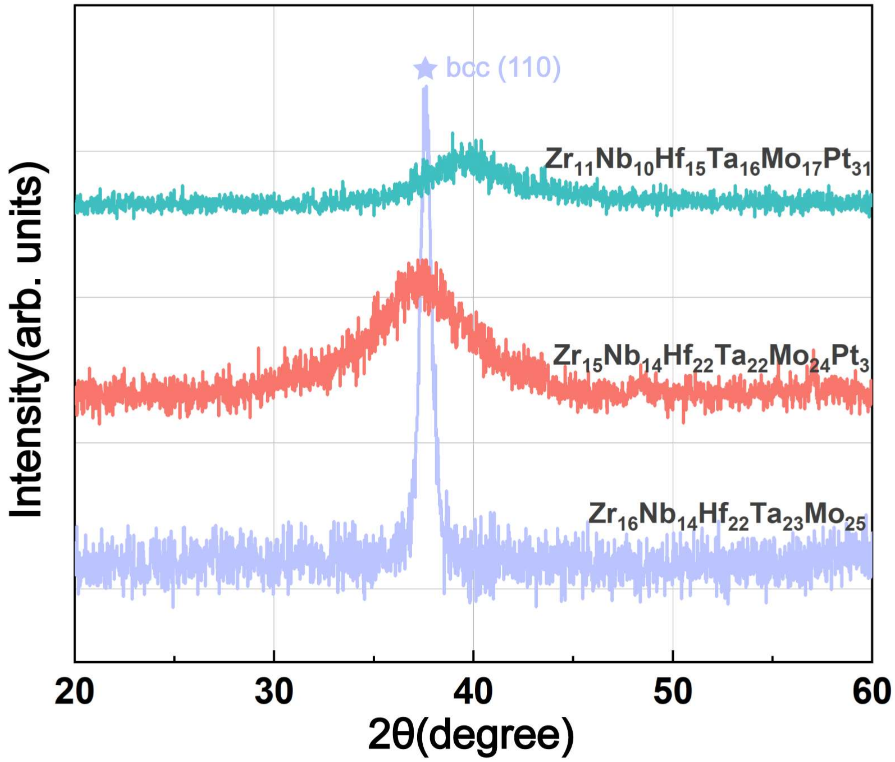

Supplementary Fig. 2 | XRD patterns of three HEAs ($Zr_{16}Nb_{14}Hf_{22}Ta_{23}Mo_{25}$, $Zr_{15}Nb_{14}Hf_{22}Ta_{22}Mo_{24}Pt_3$ and $Zr_{11}Nb_{10}Hf_{15}Ta_{16}Mo_{17}Pt_{31}$). The sharp diffraction peak corresponding to the body center cubic (bcc) (110) plane in the $Zr_{16}Nb_{14}Hf_{22}Ta_{23}Mo_{25}$ HEA changes into the "amorphous-characteristic" hump peaks in the $Zr_{15}Nb_{14}Hf_{22}Ta_{22}Mo_{24}Pt_3$, $Zr_{11}Nb_{10}Hf_{15}Ta_{16}Mo_{17}Pt_{31}$ HEAs.

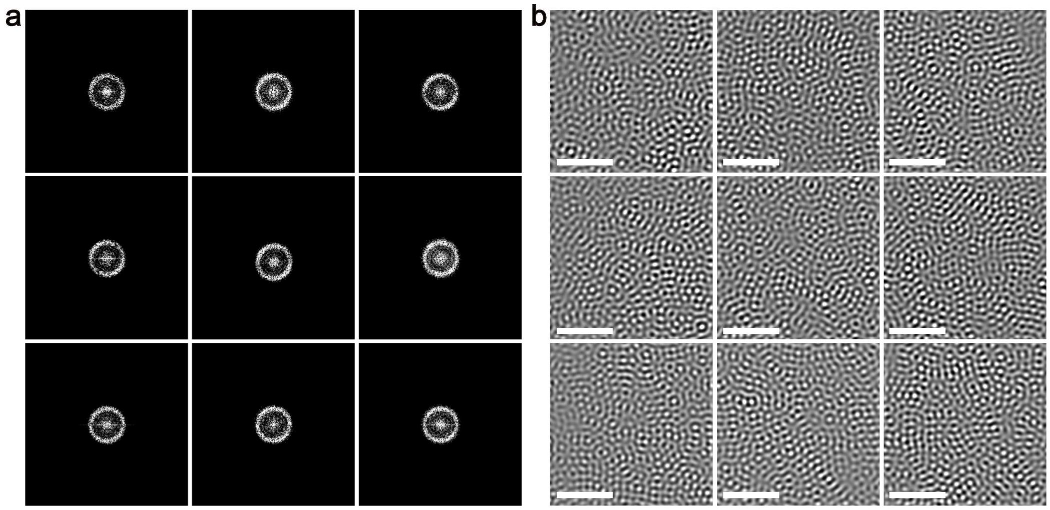

Supplementary Fig. 3 | FFT and the corresponding inverse FFT (IFFT) images taken from the divided HRTEM image in Fig. 1e. Each sub-image in **a** and **b** having 1356×1356 pixels and corresponding to a region with the dimension of 5.808× 5.808 nm^2. Scale bar in **b** is 2 nm.

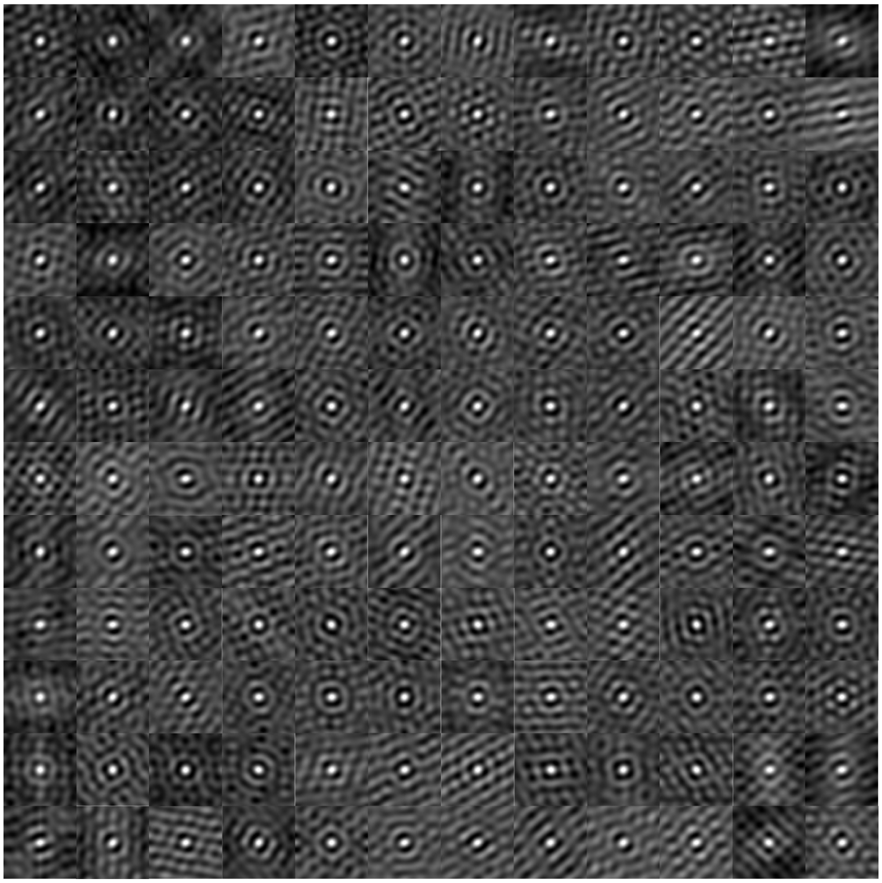

Supplementary Fig. 4 | Representative autocorrelation function (ACF) statistical analysis of Fig. 1e. The corresponding ACF-processed 1.452×1.452 nm^2 images.

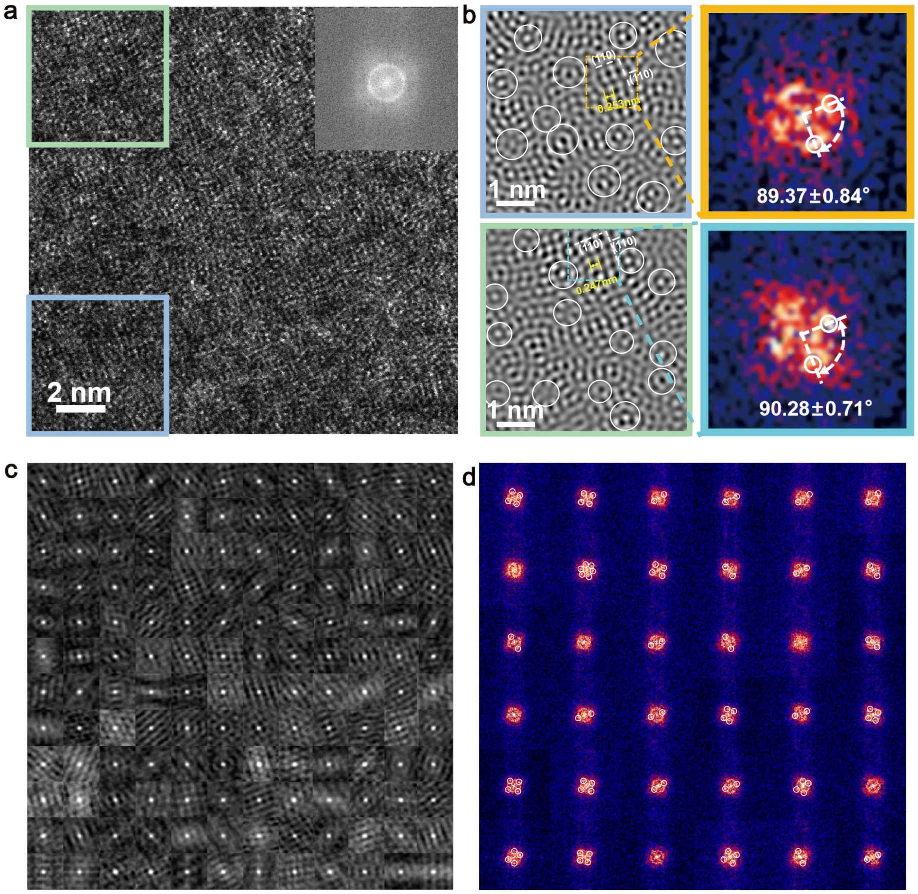

Supplementary Fig. 5 | The structural characterization and analysis of paracrystalline $Zr_{15}Nb_{14}Hf_{22}Ta_{22}Mo_{24}Pt_3$ HEA. a, The atomic-resolution HAADF-STEM images of the paracrystalline $Zr_{15}Nb_{14}Hf_{22}Ta_{22}Mo_{24}Pt_3$ HEA. **b**, Zoom-in images are taken from the blue and green squared areas, respectively, revealing the bottom-up homogeneity of the paracrystalline structure. FFT patterns corresponding to the cyan and orange squares (1.391 × 1.391 nm^2) suggest the existence of crystalline medium-range order (MRO) motifs. Disordered groups are marked by white circles. **c**, **d**, the corresponding autocorrelation function (ACF)-processed and FFT-processed images, respectively.

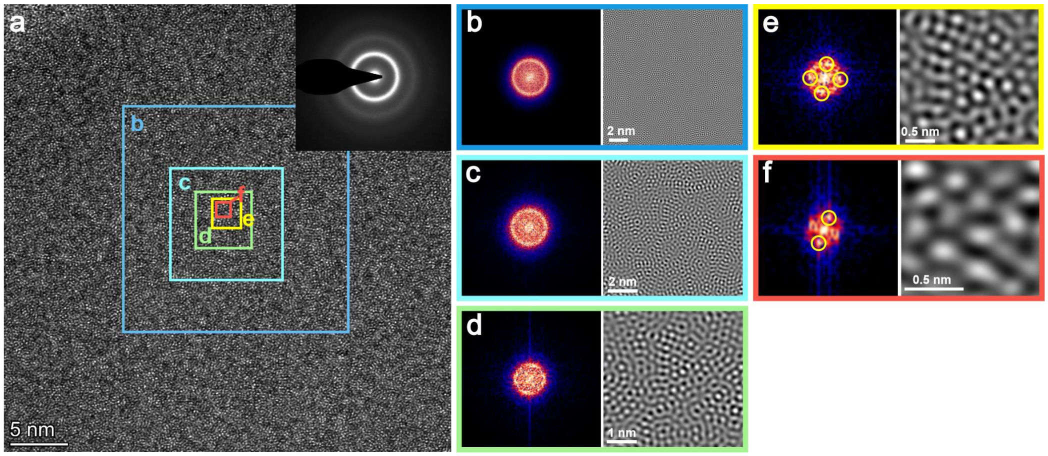

Supplementary Fig. 6 | The changing FFT characteristics with the zoom-out selected area in the HRTEM image of $Zr_{15}Nb_{14}Hf_{22}Ta_{22}Mo_{24}Pt_3$ sample. **a**, Selected area electron diffraction (SAED) pattern in the inset revealing the typical amorphous feature. **b**, **c**, The diffuse halo rings remain in the FFT-processed 19.394×19.394 nm^2 and 9.697×9.697 nm^2 patterns taken from the squared blue and cyan areas in **a**. **d-f**, Further zooming out the area of FFT-processed patterns results in the transition from discrete halo rings (4.484×4.484 nm^2 in **d**) to bright spots coupled with discrete halo rings (2.242×2.242 nm^2 in **e**), then to well-defined bright spots (1.121×1.121 nm^2 having the comparable size with medium-range order (MRO) in **f**).

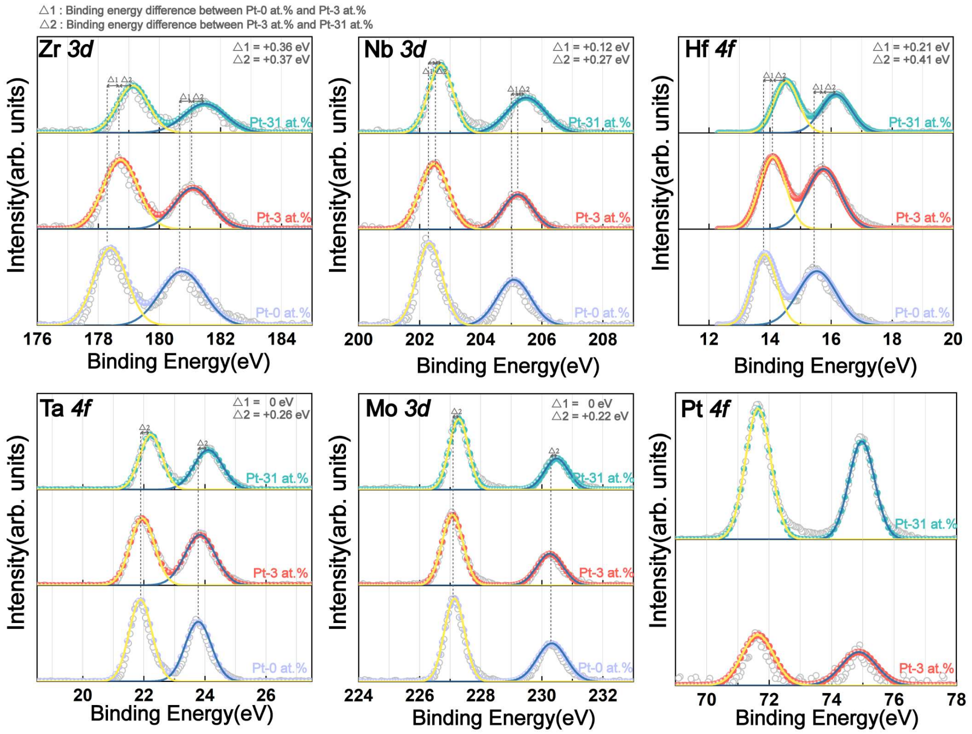

Supplementary Fig. 7 | XPS core level spectra of Zr *3d*, Nb *3d*, Hf *4f*, Ta *4f*, Mo *3d*, Pt *4f* performed on three HEAs (Pt-0 at.%, Pt-3 at.%, Pt-31 at.%). The largest Δ1 and Δ2 of Zr *3d* in Pt-0 at.% suggest the preferential formation of Pt-Zr pair.

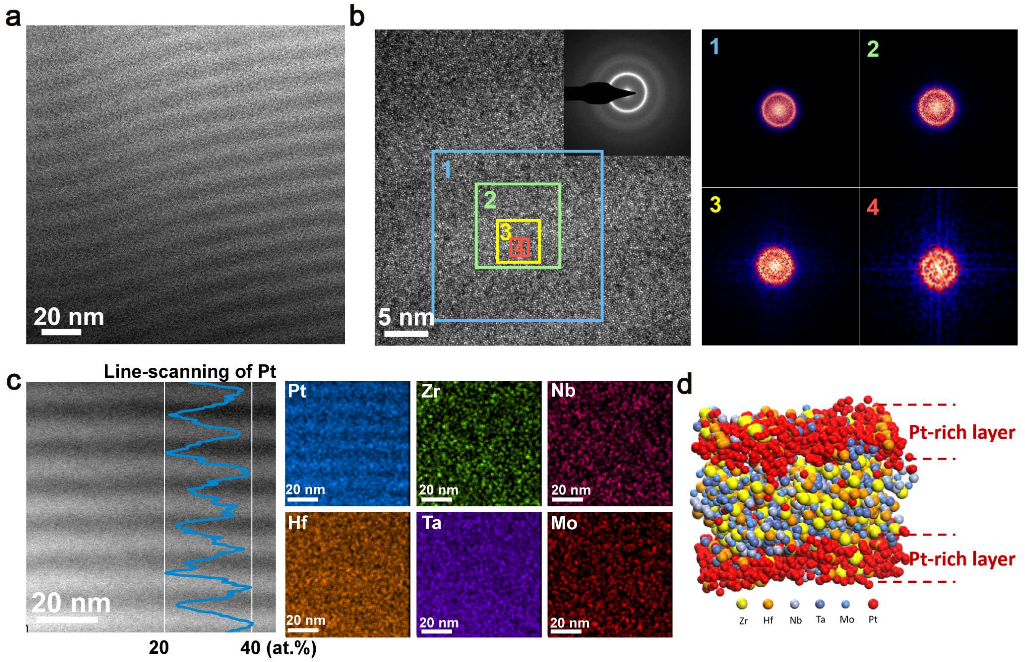

Supplementary Fig. 8 | Structural characterization and illustration of $Zr_{11}Nb_{10}Hf_{15}Ta_{16}Mo_{17}Pt_{31}$ HEA. a, HRTEM image shows the multi-layered feature and each layer is amorphous. **b**, Inset is the corresponding selected area electron diffraction (SAED) pattern of the HRTEM image showing diffuse diffraction rings. The diffuse halo rings in the corresponding FFT patterns taken from the different areas of squares in the HRTEM image (marked as 1-4) maintain unchanged, even the area of the FFT-processed pattern is down to 1.208×1.208 nm^2, suggesting the loss of crystalline medium-range order (MRO). **c**, EDS maps of each constituent element suggest that the periodic multilayer structure is distinguished by the compositional variation alternating Pt-rich and Pt-lean nanolayers. Inset is EDS line-scanning analysis of element Pt. **d**, Illustration of the multi-layered structure of $Zr_{11}Nb_{10}Hf_{15}Ta_{16}Mo_{17}Pt_{31}$ HEA.

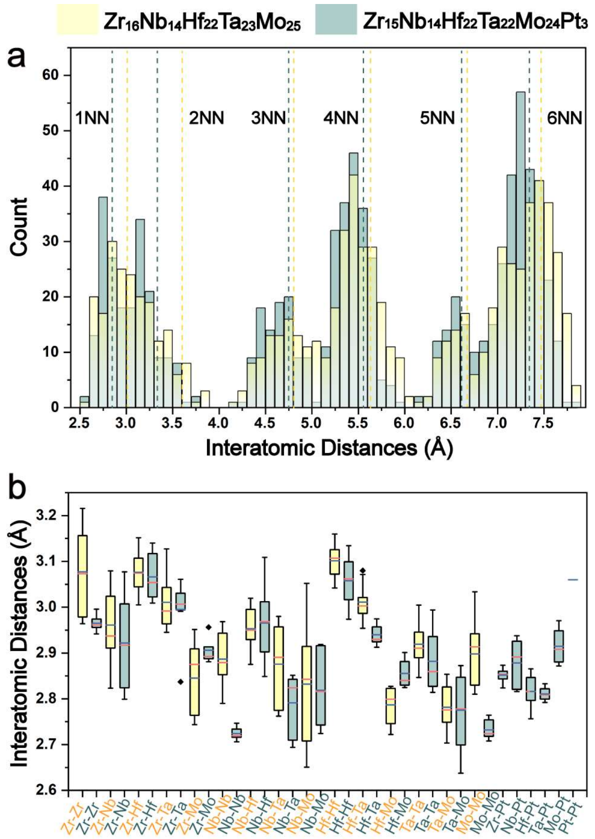

Supplementary Fig. 9 | Predictions of the interatomic distances for Zr-Nb-Hf-Ta-Mo and Zr-Nb-Hf-Ta-Mo-Pt HEAs via first-principles calculations. a, Distributions of the first to sixth nearest neighbors (NN) interatomic distances. The comparison of distributions of interatomic distances calculated by Special Quasi-random Structures (SQS) shows the more lattice distortion for Zr-Nb-Hf-Ta-Mo-Pt HEA. The yellow (blue) dashed lines indicate the average interatomic distances for the first to sixth NN of the Zr-Nb-Hf-Ta-Mo (Zr-Nb-Hf-Ta-Mo-Pt) HEAs. **b**, The box plot shows the distribution of interatomic distances of 1NN bonds in the SQSs. The yellow boxes correspond to the Zr-Nb-Hf-Ta-Mo HEA, and the blue boxes represent the Zr-Nb-Hf-Ta-Mo-Pt HEA. The box indicates the range from the first and third quantiles, the whisker extends to 1.5 times the interquartile ranges, and the dots represent outliers beyond the whiskers. Median and mean lines are represented by blue and red lines, respectively.

The distributions of the first through sixth nearest neighbor atomic distances for Zr-Nb-Hf-Ta-Mo and Zr-Nb-Hf-Ta-Mo-Pt HEAs have been displayed in Supplementary Fig. 9a. The blue and yellow regions represent the distribution of nearest neighbor distances for Zr-Nb-Hf-Ta-Mo and Zr-Nb-Hf-Ta-Mo-Pt, respectively. The average first, second, third, fourth, fifth and sixth nearest neighbor distances are 3.04, 3.60, 4.80, 5.63, 6.67, and 7.47 Å for ZrNbHfTaMo and 2.88, 3.34, 4.73, 5.55, 6.63, and 7.37 Å for ZrNbHfTaMoPt, respectively, which are marked by the blue and yellow dashed lines in Supplementary Fig. 9a. It is worth noting that the obvious change in the state of distribution of neighbor distances with the addition of the Pt element to the Zr-Nb-Hf-Ta-Mo system, indicating an increase in the lattice distortion. In addition, the atomic-species pairs in Supplementary Fig. 9b suggest the distribution of the first nearest neighbor (1NN) bond lengths. It is apparent that the addition of Pt, an element with a relatively smaller atomic radius relative to the other constituent elements, leads to a decrease in the range of 1NN bond lengths for most of the atomic-specie pairs in the Zr-Nb-Hf-Ta-Mo system.

Furthermore, the lattice-distortion factor, $\overline{u}^D$, has been introduced to visualize the degree of lattice distortion in Zr-Nb-Hf-Ta-Mo and Zr-Nb-Hf-Ta-Mo-Pt HEAs[1]. $\overline{u}^D$ can be expressed as:

$$\overline{u}^D = \sqrt{\sum_i^n (d_i^{eff} - \overline{d})^2 \Big/ n} \quad (1)$$

where d_i^{eff} is the effective interatomic distance of the ith element, and $\overline{d}$ is average interatomic distance of *n* elements, which are determined from the lattice constant of the incorporated elements associated with their respective crystal structure, and *n* is the number of constituent elements in HEAs. Therefore, a 50-atom Zr-Nb-Hf-Ta-Mo HEA model with equiatomic ratios has been built via SQS. To testify to the influence of Pt on lattice distortion, a 50-atom Zr-Nb-Hf-Ta-Mo-Pt HEA model in which five atomic sites were equipped by Pt atoms has also been built via SQS. The calculated values of

$\bar{u}^D$ for $Zr_{20}Nb_{20}Hf_{20}Ta_{20}Mo_{20}$ and $Zr_{18}Nb_{18}Hf_{18}Ta_{18}Mo_{18}Pt_{10}$ HEAs are 0.2082 and 1.2726, respectively, manifesting the significant increment in average lattice distortion by adding Pt. It indicates that the addition of the Pt element in the Zr-Nb-Hf-Ta-Mo HEA effectively promotes the large variation of interatomic distances, which results in the significant increase of lattice distortion and contributes to the local amorphization induced by Pt.

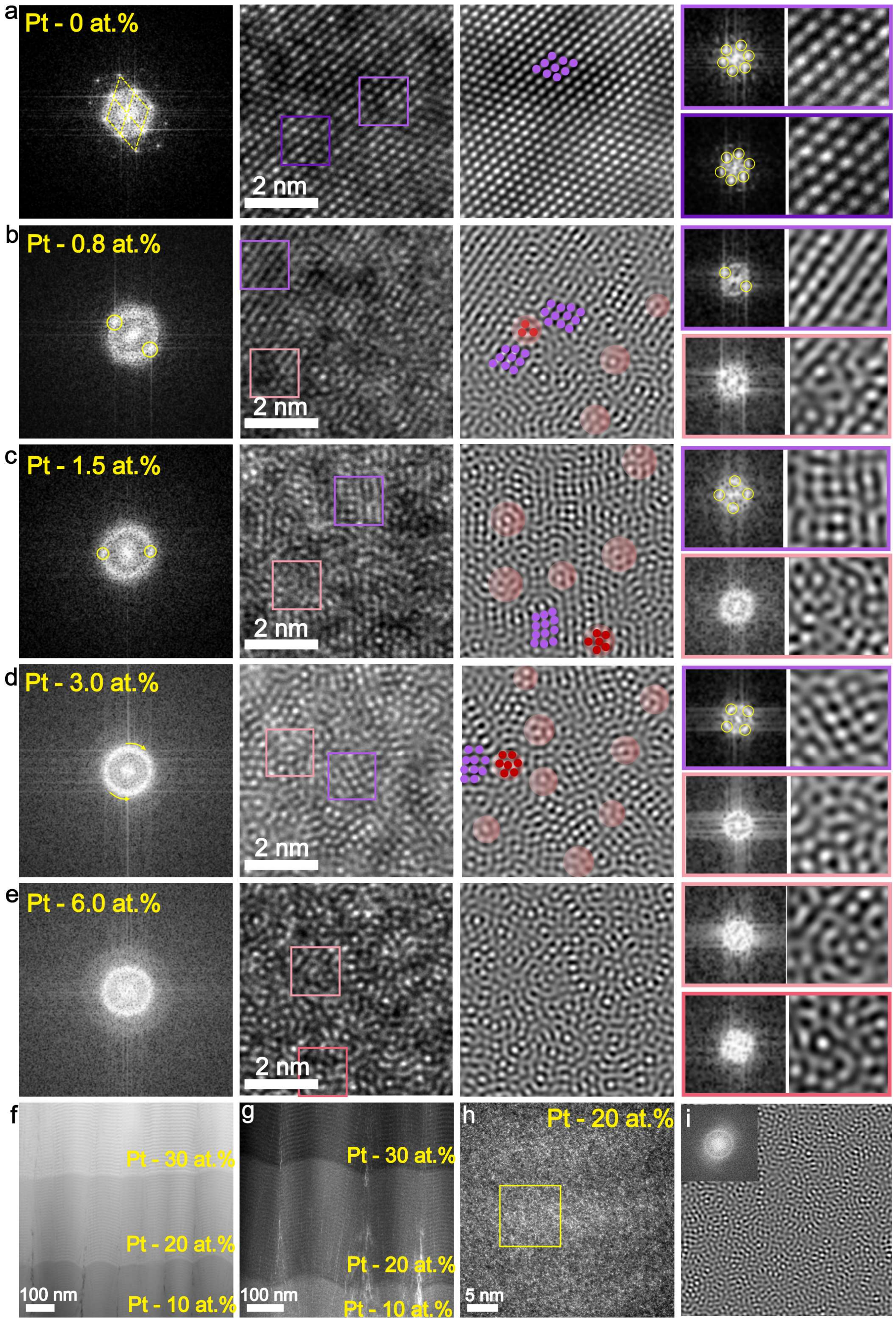

Supplementary Fig. 10 | Microstructure evolution of the compositionally-graded sample. Yellow circles in FFT patterns represent the bright "diffraction" spots, revealing the ordered arrangement. The pink circles

in inverse FFT (IFFT) images represent the disordered groups and the purple dots represent the atoms with ordered arrangement. **a**, FFT pattern, HRTEM and the corresponding IFFT images of Pt- 0 at.% HEA. Two local regions in the HRTEM image (marked as purple squares) are selected for magnification and FFT processing. **b-d**, FFT pattern, HRTEM and the corresponding IFFT images of Pt-0.8 at.%, Pt-1.5 at.%, Pt-3.0 at.% HEA, respectively. The purple and pink regions in the HRTEM image are selected for magnification and FFT processing, representing ordered and disordered arrangement respectively. **e**, FFT pattern, HRTEM and the corresponding IFFT images of Pt- 6.0 at.% HEA. Two local regions in the HRTEM image (marked as pink squares) are selected for magnification and FFT processing. With the further increment of disordered groups by 3 at. % Pt, the paracrystalline structure emerges and it is completely transformed into the fully amorphous structure in the 6 at. % Pt HEA. **f**, **g**, The STEM and HRTEM images containing Pt-10 at.%, Pt-20 at.%, Pt-30 at.% HEA. In the HEA with higher Pt content (≥10 at. %), the nano-multilayered architecture with alternating Pt-lean/rich amorphous nanolayers can be self-assembled owing to phase separation. **h**, HRTEM image of Pt-20 at.% HEA. **i**, IFFT image taken from the yellow square in **h**, indicating the amorphous characteristics of the nano-multilayer structure. Inset is the corresponding FFT pattern.

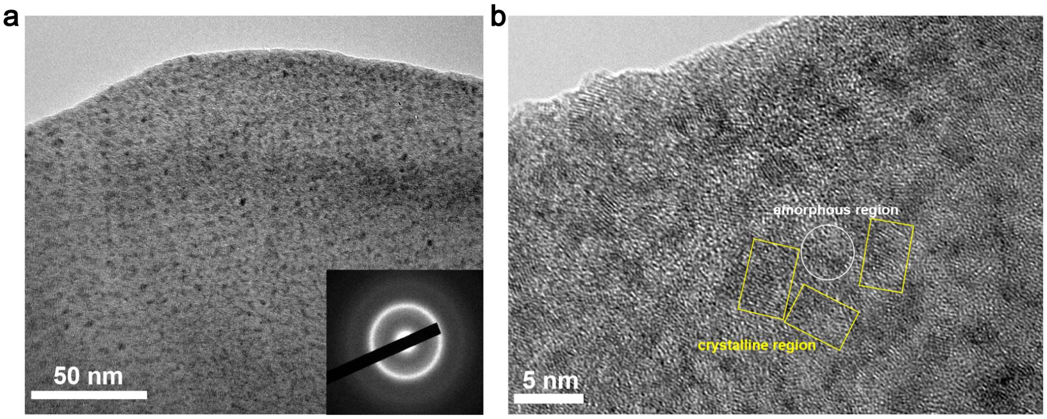

Supplementary Fig. 11 | Structural characterization of $Zr_{15}Nb_{14}Hf_{19}Ta_{20}Mo_{24}Au_{8}$ HEA. a, **b**, TEM and HRTEM images exhibit the array-like nanocrystal-glass dual-phase structure. Inset in **a** is the corresponding selected area electron diffraction (SAED) pattern showing diffuse diffraction rings, manifesting the Au-induced crystalline-to-amorphous transition. Unlike Pt-induced paracrystalline structure, the nanocrystals above 2 nm size are retained because the ΔH_{mix} between Au and other elements is not as high as that of Pt.

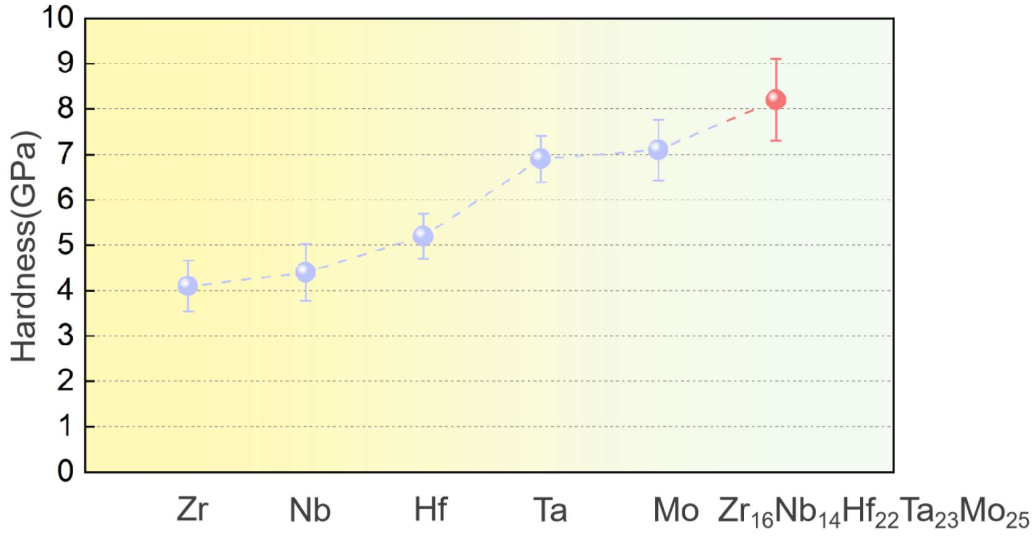

Supplementary Fig. 12 | Hardness of $Zr_{16}Nb_{14}Hf_{22}Ta_{23}Mo_{25}$ HEA and each corresponding constituent sample. Error bars represent standard deviation.

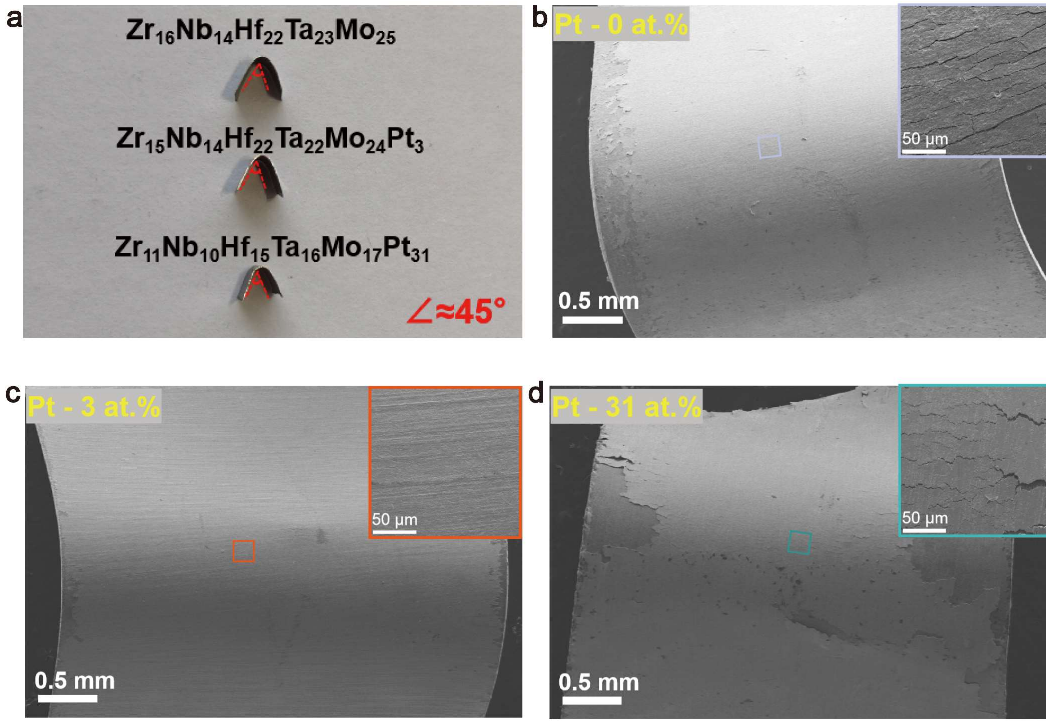

Supplementary Fig. 13 | Simple bending tests. a, The simple bend tests with a 45° bending angle for three HEA films deposited on Ti foils, manifesting their plasticity. **b-d**, The corresponding surface SEM images on the bent region for Pt-free, Pt-3%, and Pt-31% HEAs, respectively, reveal the distinct plasticity in the Pt-3% paracrystalline HEA, evidenced by the invisible cracks and outstanding surface integrity.

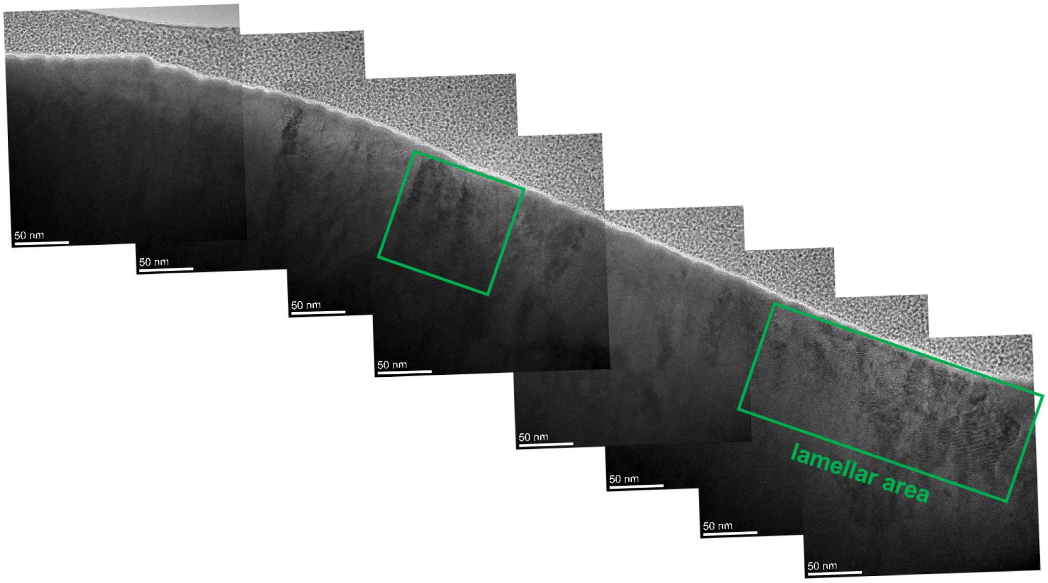

Supplementary Fig. 14 | HRTEM images of the deformed region under indenter in $Zr_{16}Nb_{14}Hf_{22}Ta_{23}Mo_{25}$ HEA. A large number of lamellar areas appear (as marked in the green square) because of the deformation-induced crystalline-to-amorphous transition.

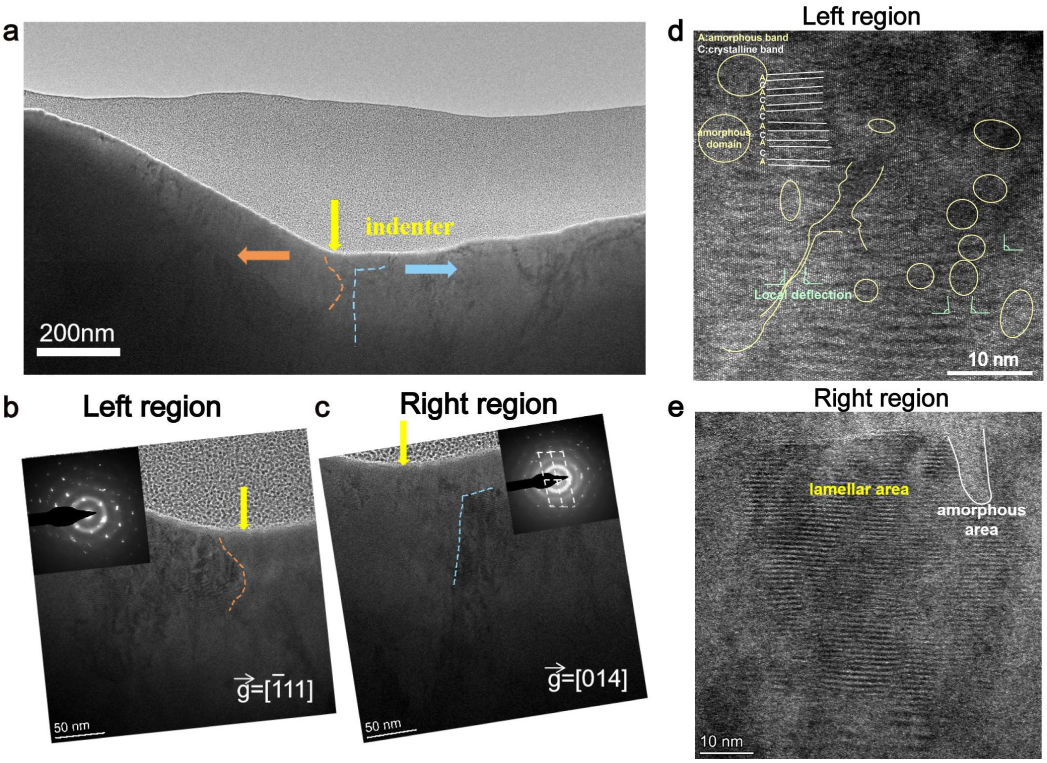

Supplementary Fig. 15 | Microstructures of overall deformation region containing differently oriented grains under indenter in the $Zr_{16}Nb_{14}Hf_{22}Ta_{23}Mo_{25}$ HEA. a, TEM image suggesting the profile of the indentation-induced deformation region. **b**, **c**, The large orientation deviation between two grains exists below the indentation. **d**, **e**, HRTEM images in left region and right region under indentation, respectively, suggest the deformation-induced crystalline-to-amorphous transition.

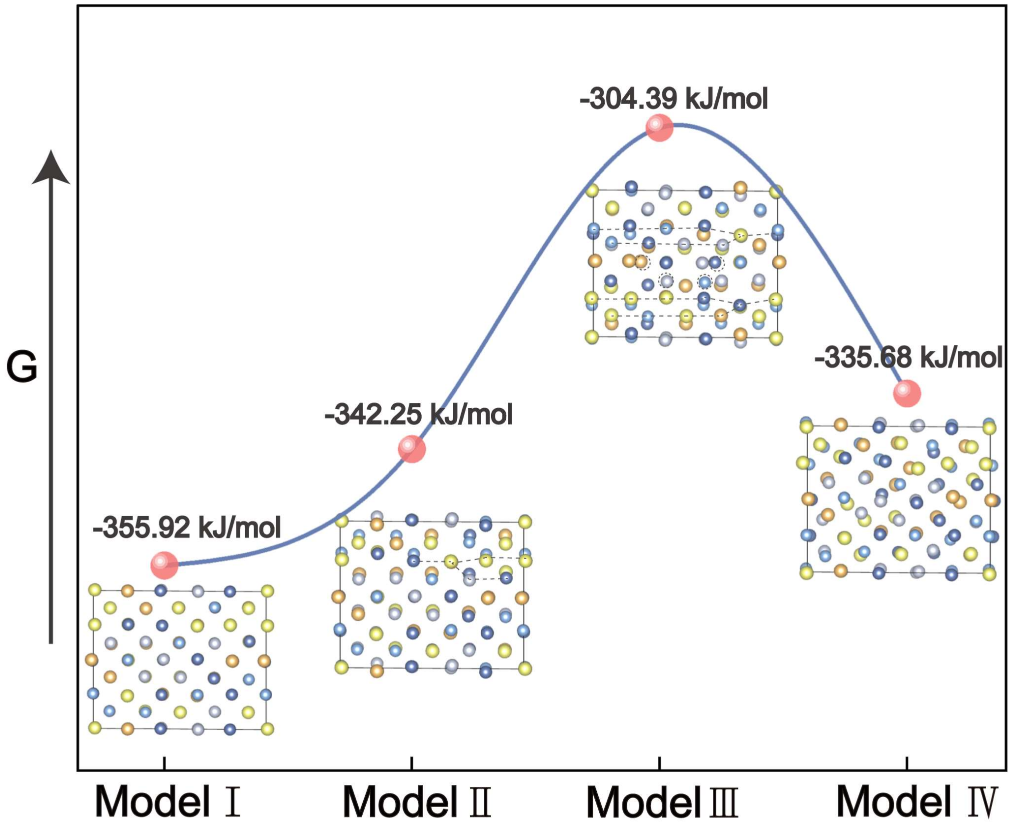

Supplementary Fig. 16 | A schematic diagram of free energy level for Zr-Nb-Hf-Ta-Mo HEA in different deformation states. Model Ⅰ: the crystalline model without defects; Model Ⅱ: the crystalline model with low-density defects (one edge dislocation); Model Ⅲ: the crystalline model with high-density defects (two edge dislocations and several point defects); Model Ⅳ: the amorphous model. The gray dashed lines and the gray dashed circles in the model diagram in the inset represent edge dislocations and point defects, respectively.

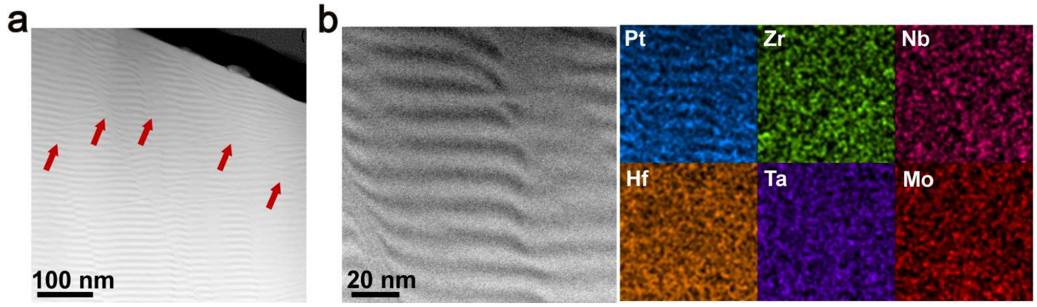

Supplementary Fig. 17 | Microstructures of deformation region under indenter in the $Zr_{11}Nb_{10}Hf_{15}Ta_{16}Mo_{17}Pt_{31}$ HEA. a, STEM image reveals the existence of much larger shear bands relative to paracrystalline HEA, as marked by the red arrows, suggesting the more strongly localized plastic flow. Generally, shear banding events accompany by the stress-driven glass-to-liquid transition in which shear stress break the atomic structure[2, 3] so that the shear banding favor the atomic diffusion process[4, 5, 6, 7]. It can be seen that the layered structure alternating with Pt-rich and Pt-lean nanolayers disappears along with the shear banding propagation because of the rapid mobility of Pt, as shown in the EDS maps in **b**.

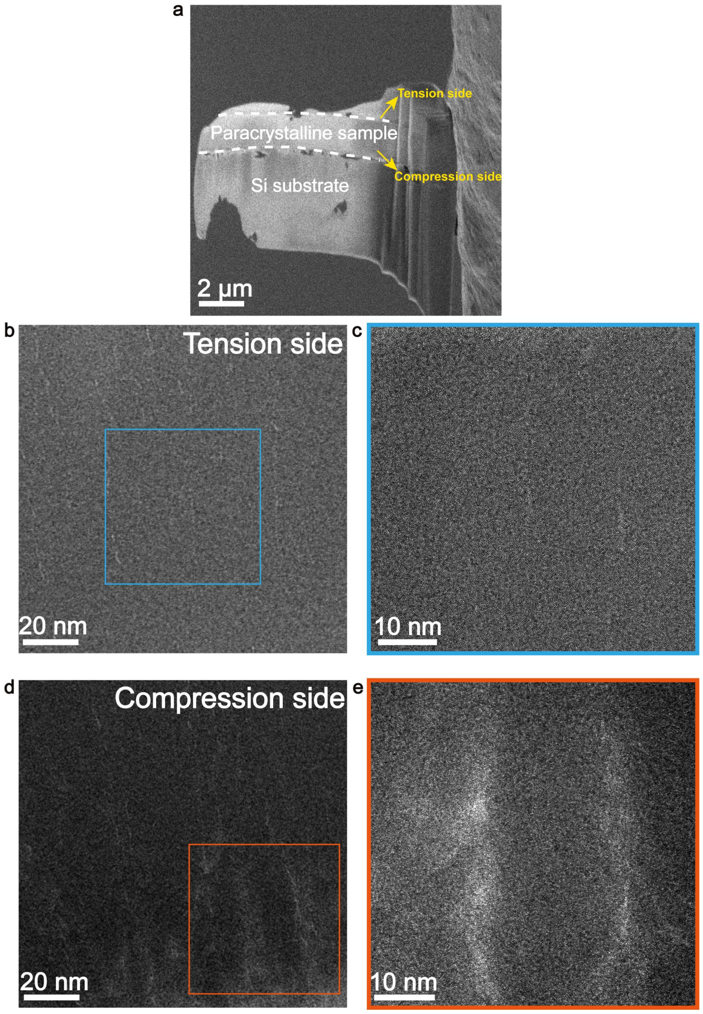

Supplementary Fig. 18 | Microstructures of bent region in the paracrystalline $Zr_{15}Nb_{14}Hf_{22}Ta_{22}Mo_{24}Pt_3$ HEA. a, SEM image of the bent sample during focused ion beam (FIB) milling. **b**, **d**, TEM images in the tension and compression regions, respectively, show the nanoscale shear bands (SBs). **c**, **e**, HRTEM images of the blue and orange squares in **b** and **d**, respectively. These nanoscale SBs with wavy trajectory can be easily distinguished from the surrounding matrix. The size of SBs in the compression region is obviously larger than that in the tension region.

Supplementary Table 1 | Atomic radius of each element involved in the present HEAs[8].

Element	Zr	Nb	Hf	Ta	Mo	Pt	Au
Atom radius (pm)	160.25	142.9	157.75	143	136.26	138.7	144.2

Supplementary Table 2 | The values of $\Delta H_{\{AB\}}^{mix}$ (kJ/mol) for atomic pairs between the consistent elements involved in the present HEAs[9].

$\Delta H_{\{AB\}}^{mix}$	Zr	Nb	Hf	Ta	Mo	Pt	Au
Zr		4	0	3	-6	-100	-74
Nb	4		4	0	-6	-67	-32
Hf	0	4		3	-4	-90	-63
Ta	3	0	3		-5	-66	-32
Mo	-6	-6	-4	-5		-28	3
Pt	-100	-67	-90	-66	-28		
Au	-74	-32	-63	-32	3		

Supplementary Table 3 | Calculated parameters ΔH_{mix}, ΔS_{mix}, δ, and Ω by average method for $Zr_{16}Nb_{14}Hf_{22}Ta_{23}Mo_{25}$, $Zr_{15}Nb_{14}Hf_{22}Ta_{22}Mo_{24}Pt_3$, $Zr_{11}Nb_{10}Hf_{15}Ta_{16}Mo_{17}Pt_{31}$, and $Zr_{15}Nb_{14}Hf_{19}Ta_{20}Mo_{24}Au_8$ HEAs, predicting the Pt/Au-induced structural evolution.

Sample	ΔH_{mix} (kJ/mol)	ΔS_{mix} (J/K·mol)	δ (%)	Ω
$Zr_{16}Nb_{14}Hf_{22}Ta_{23}Mo_{25}$	-3.86	13.18	6.38	9.37
$Zr_{15}Nb_{14}Hf_{22}Ta_{22}Mo_{24}Pt_3$	-18.43	13.89	6.37	2.05
$Zr_{11}Nb_{10}Hf_{15}Ta_{16}Mo_{17}Pt_{31}$	-116.55	14.27	6.05	0.31
$Zr_{15}Nb_{14}Hf_{19}Ta_{20}Mo_{24}Au_8$	-25.55	14.49	6.17	1.48

Supplementary References:

1. Lee C*, et al.* Lattice-Distortion-Enhanced Yield Strength in a Refractory High-Entropy Alloy. *Adv Mater*, e2004029 (2020).

2. Todd C. Hufnagel a, Christopher A. Schuh b, **, Michael L. Falk a, ***. Deformation of metallic glasses: Recent developments in theory, simulations, and experiments. *Acta Materialia*, (2016).

3. Guan P, Chen M, Egami T. Stress-temperature scaling for steady-state flow in metallic glasses. *Phys Rev Lett* **104**, 205701 (2010).

4. Ngai KL, Bin Yu H. Origin of ultrafast Ag radiotracer diffusion in shear bands of deformed bulk metallic glass Pd40Ni40P20. *Journal of Applied Physics* **113**, (2013).

5. Bokeloh J, Divinski SV, Reglitz G, Wilde G. Tracer Measurements of Atomic Diffusion inside Shear Bands of a Bulk Metallic Glass. *Physical Review Letters* **107**, (2011).

6. Liu S*, et al.* Deformation-enhanced hierarchical multiscale structure heterogeneity in a Pd-Si bulk metallic glass. *Acta Materialia* **200**, 42-55 (2020).

7. Schuh C, Hufnagel T, Ramamurty U. Mechanical behavior of amorphous alloys. *Acta Materialia* **55**, 4067-4109 (2007).

8. Miracle DB, Senkov ON. A critical review of high entropy alloys and related concepts. *Acta Materialia* **122**, 448-511 (2017).

9. Akira Takeuchi AI. Classification of Bulk Metallic Glasses by Atomic Size Difference, Heat of Mixing and Period of Constituent Elements and Its Application to Characterization of the Main Alloying Element. *Mater Trans* **46**, 2817-2829 (2005).
