# Supplementary material for: Pt-induced atomic-level tailoring towards paracrystalline high-entropy alloy
Source: Nat Commun. 2023 Feb 11;14:775. doi: 10.1038/s41467-023-36423-1 (PMC9922268; doi:10.1038/s41467-023-36423-1)
Supplement: Supplementary file 2 — Description of Additional Supplementary Files [file 41467_2023_36423_MOESM2_ESM.pdf]

Description of Additional Supplementary Files

File Name: Supplementary Movie 1
Description: In situ SEM pillar compression test and engineering stressstrain curve of Pt-0 at.% HEA

File Name: Supplementary Movie 2
Description: In situ SEM pillar compression test and engineering stressstrain curve of Pt-3 at.% HEA

File Name: Supplementary Movie 3
Description: In situ SEM pillar compression test and engineering stressstrain curve of Pt-31 at.% HEA
